# Functional Identification of Catalytic Metal Ion Binding Sites within RNA

**DOI:** 10.1371/journal.pbio.0030277

**Published:** 2005-08-16

**Authors:** James L Hougland, Alexander V Kravchuk, Daniel Herschlag, Joseph A Piccirilli

**Affiliations:** **1**Department of Chemistry, University of Chicago, Illinois, United States of America,; **2**Department of Biochemistry, Stanford University, California, United States of America,; **3**Department of Biochemistry and Molecular Biology, University of Chicago, Illinois, United States of America,; **4**Howard Hughes Medical Institute, University of Chicago, Illinois, United States of America; Department of Molecular Biology, Scripps Research InstituteUnited States of America

## Abstract

The viability of living systems depends inextricably on enzymes that catalyze phosphoryl transfer reactions. For many enzymes in this class, including several ribozymes, divalent metal ions serve as obligate cofactors. Understanding how metal ions mediate catalysis requires elucidation of metal ion interactions with both the enzyme and the substrate(s). In the *Tetrahymena* group I intron, previous work using atomic mutagenesis and quantitative analysis of metal ion rescue behavior identified three metal ions (M_A_, M_B_, and M_C_) that make five interactions with the ribozyme substrates in the reaction's transition state. Here, we combine substrate atomic mutagenesis with site-specific phosphorothioate substitutions in the ribozyme backbone to develop a powerful, general strategy for defining the ligands of catalytic metal ions within RNA. In applying this strategy to the *Tetrahymena* group I intron, we have identified the *pro-S*_P_ phosphoryl oxygen at nucleotide C262 as a ribozyme ligand for M_C_. Our findings establish a direct connection between the ribozyme core and the functionally defined model of the chemical transition state, thereby extending the known set of transition-state interactions and providing information critical for the application of the recent group I intron crystallographic structures to the understanding of catalysis.

## Abstract 2

A combination of substrate atomic mutagenesis with site-specific substitutions in the ribozyme backbone allow the ligands of catalytic metal ions to be identified.

## Introduction

Phosphoryl transfer reactions occur ubiquitously in biology, playing roles in gene replication, recombination, and expression. To orchestrate these central biological processes, biomacromolecules have harnessed the catalytic power of divalent metal ions [1–10]. The chemistry underlying these cellular events hinges critically on the metal ion interactions that occur during function. However, there exists little functional data defining the coordination environment of individual metal ions during catalysis and correspondingly few methods for obtaining such information.

Structural analyses can serve as a powerful starting point for investigation of catalytic metal ions, but these approaches provide no direct information about transition-state interactions. Reflecting this uncertainty, for a given enzyme or enzyme family the number of active-site metal ions and their proposed interactions during catalysis can vary with both enzyme and observer ([2–10] and references therein). For example, restriction enzymes have been crystallized with zero, one, two, or three metal ions in their active sites [7,8,10], and different RNase H crystal structures support either a one or two metal ion mechanism [3]. This variability in metal ion binding also occurs for RNA. In structures of the hammerhead ribozyme and tRNA, Mg^2+^ and Mn^2+^ occupy different sites [11,12], and recent group I intron structures exhibit differences in the number, location, and identity of active-site metal ions [13–15]. Thus, only the combination of structural and functional studies can establish metalloenzyme mechanisms unambiguously.

To understand how metalloenzymes utilize the catalytic power of metal ions, we must identify individual catalytic metal ions functionally, determine their relationships to each other, define their coordination environment, and establish the network of interactions that position the coordinating groups. Metal ion rescue experiments using substrate mutations offer a strategy to identify specific catalytic metal ion interactions within enzyme active sites, a particularly formidable challenge in ribozymes due to the sea of metal ions that interact electrostatically with the anionic phosphodiester backbone. Functionally deleterious sulfur or nitrogen perturbations that exhibit rescue upon increasing cation softness (e.g., replacing Mg^2+^ with Cd^2+^ or Mn^2+^) suggest direct metal ion coordination during catalysis [16,17]. This approach has revealed catalytic metal ion interactions with enzyme substrates in the *Tetrahymena* group I ribozyme [18–21], the ai5γ group II intron [22,23], RNase P [24], the hammerhead ribozyme [25,26], the human spliceosome [27,28], and many protein enzymes (e.g., [16,29–32] and references therein).

The *Tetrahymena* group I ribozyme catalyzes nucleotidyl transfer from an oligonucleotide substrate that mimics the natural 5′-splice site to an exogenous guanosine (G) that serves as the nucleophile in a reaction analogous to the first step of group I intron self-splicing (Equation 1) [33,34].



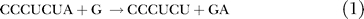



**Figure 1:**
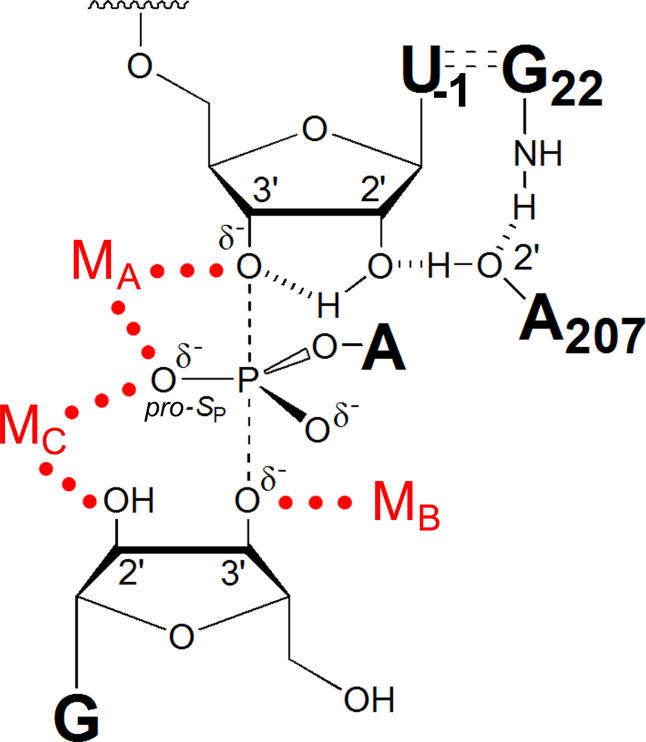
Model of the *Tetrahymena* Ribozyme Transition State during the First Step of Splicing The three identified catalytic metal ions (M_A_, M_B_, and M_c_) and their transition-state interactions with the U(-1) 3′-oxygen, G nucleophile 3′-oxygen, and G 2′-OH, respectively, along with the M_A_ and M_C_ interactions with the scissile phosphate *pro*-*S*_P_ oxygen, are shown by red dots [35,36]. The 2′-OH of U(-1) participates in a hydrogen-bonding network with the 2′-OH of A207 and the exocyclic amine of the G·U wobble pair [58,72] and donates a hydrogen bond to the adjacent 3′-oxygen in the transition state [71]; the hydrogen bonds are shown as hashed lines.

Metal ion rescue experiments have identified four atoms within the oligonucleotide substrate and G nucleophile that interact with metal ions in the chemical transition state [18–21]. To determine whether one or several distinct metal ions mediate these interactions, Shan et al. developed thermodynamic fingerprint analysis, quantitatively analyzing the reactivity of modified substrates relative to unmodified substrates over a range of rescuing metal ion concentrations [35]. In this approach, the reactions for both modified and native substrates start from the same ground state and monitor the same elementary reaction steps. The resulting rescue profiles serve as distinctive “fingerprints” for the rescuing metal ion(s), revealing by comparison whether the same or distinct metal ions interact with the identified substrate ligands. Thermodynamic fingerprint analysis and related analyses [36] using a series of substrates bearing single or multiple atomic perturbations have provided functional evidence for a network of three distinct metal ions within the *Tetrahymena* ribozyme active site (Figure 1), making a total of five interactions with the reaction's transition state. Metal ions coordinate to the 3′-oxygen leaving group (M_A_), the 3′-oxygen on the G nucleophile (M_B_), and the 2′-hydroxyl of the G nucleophile (M_C_). Two of these metal ions (M_A_ and M_C_) also contact the *pro*-*S*_P_ oxygen of the scissile phosphate [35,36]. However, the ligands within the ribozyme core architecture that bind and position these metal ion cofactors remain largely unknown, leaving the catalytic metal ion coordination environments undefined.

The non-bridging phosphate oxygens of the RNA backbone commonly serve as ligands for divalent metal ions. For the *Tetrahymena* group I ribozyme and other RNA enzymes, phosphorothioate interference studies have generated a plethora of ligand candidates for metal ions [17,26,37–52]. However, there have been few attempts to link these putative ligands to metal ions directly involved in catalysis [42,53,54]. Using the *Tetrahymena* group I ribozyme as a model system, we have combined thermodynamic fingerprint analysis with an array of atomically perturbed substrates and ribozyme site- and stereo-specific phosphorothioate mutations to develop a general functional approach for identifying ligands for the catalytic metal ions. Our findings establish a direct connection between the ribozyme core and the functionally defined model of the chemical transition state, thereby providing information critical for the application of the recent group I intron crystallographic structures to the understanding of catalysis.

## Results

### Choosing Sites for Phosphorothioate Substitution within the Ribozyme Core

**Figure 2:**
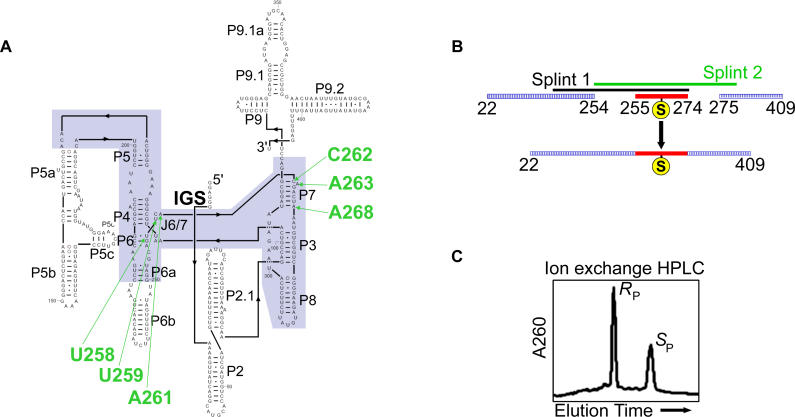
Construction of Ribozymes Containing Site-Specific Phosphorothioate Substitutions (A) Secondary structure of the *Tetrahymena* group I ribozyme. The ribozyme conserved core is highlighted in blue, and the six positions of phosphorothioate substitution within the J6/7 and P7 regions are labeled in green. The internal guide sequence at the 5′-terminus of the ribozyme is labeled “IGS.” (B) Ligation strategy for constructing mutant ribozymes. Following ion exchange HPLC purification of the phosphorothioate diastereomers (C), the phosphorothioate-substituted mutation oligonucleotides were ligated into full-length ribozymes by two successive-splint mediated ligations using T4 DNA ligase [77], as described in Materials and Methods *.*

Backbone mutation sites were chosen prior to the release of the recently reported group I intron structures [13–15]. To guide our choice of substitution sites, we focused on previously reported interferences arising from random *R*_P_-phosphorothioate incorporation into the phylogenetically conserved core regions of the *Tetrahymena* group I intron. As Mg^2+^ coordinates poorly to sulfur, the *R*_P_-phosphorothioate interferences could reflect direct disruption of a metal ion interaction with the *pro*-*R*_P_ phosphate oxygen, indirect disruption of a metal interaction with the geminal *pro*-*S*_P_ phosphate oxygen, or other effects. A literature survey identified 14 sites of *R*_P_-phosphorothioate interference within the ribozyme's conserved core [17,47,52,55]. Herein we analyze ribozymes containing site-specific phosphorothioate incorporation at six sites within the J6/7 and P7 regions of the ribozyme (Figure 2A). We constructed these mutant ribozymes semi-synthetically with both *R*_P_- and *S*_P_-phosphorothioate mutations at these six sites (Figure 2B), resulting in 12 variant ribozymes.

**Figure 3:**
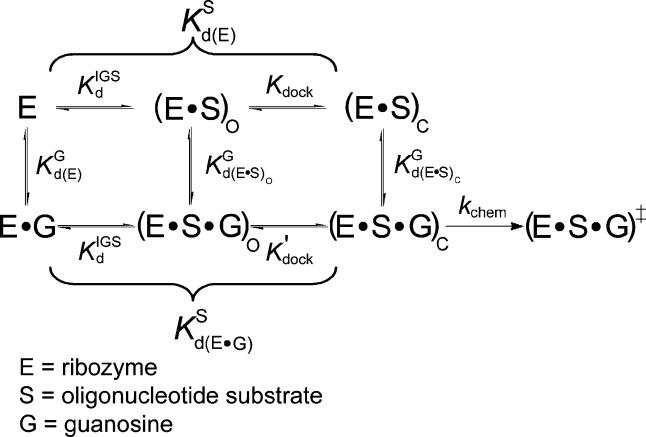
The *Tetrahymena* Ribozyme Reaction Pathway The ribozyme binds the oligonucleotide substrate (in two steps) and the exogenous G that serves as the nucleophile in the ribozyme reaction as described in the text ([33,56,57] and references therein). *K* ^G^_d(E)_, *K* ^G^_d(E·S)_O__, and *K* ^G^_d(E·S)_c__ are G dissociation constants from free E, (E·S)_O_, and (E·S)_C_, respectively. *K* ^IGS^_d_ is the dissociation constant for the oligonucleotide substrate from the internal guide sequence, and *K*_dock_ and *K*′_dock_ are the docking equilibria for the E·S and E·S·G complexes, respectively. *K* ^S^_d(E)_ and *K* ^S^_d(E·G)_ are the observed dissociation constants for the oligonucleotide substrate from free E and the E·G complex, respectively, and *k*_chem_ is the observed rate of oligonucleotide substrate cleavage.

The variant ribozymes were characterized kinetically within the known framework of the *Tetrahymena* ribozyme reaction (Figure 3; [33,56,57] and references therein). The oligonucleotide substrate (S; Table 1) binds to the ribozyme (E) in two steps. First, S forms Watson–Crick base pairs with the ribozyme's internal guide sequence (see Figure 2A) to give the open complex (E·S)_O_. The resulting P1 helix then “docks” into the ribozyme core via tertiary interactions, forming the closed complex (E·S)_C_ ([33,57–59] and references therein). G binds to give the ternary (E·S·G)_C_ complex, and the reaction proceeds through the phosphoryl transfer step (*k*_chem_), resulting in cleavage of the oligonucleotide substrate.

**Table 1:**
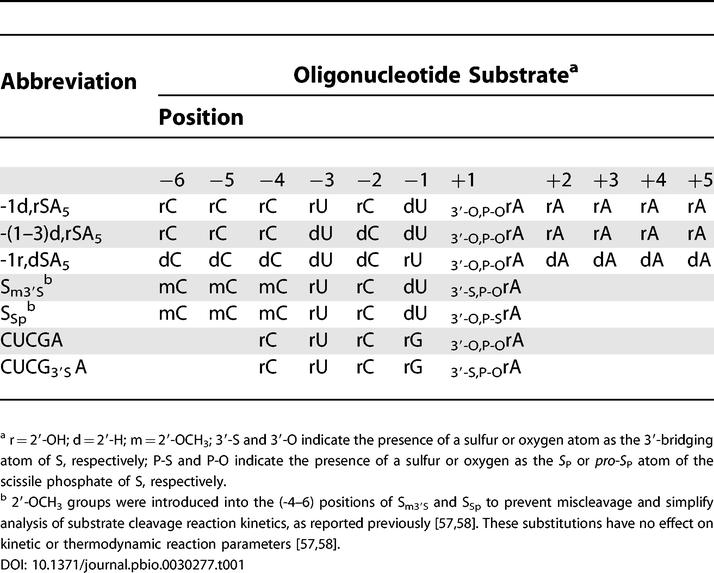
Oligonucleotide Substrates Used Herein

**Figure 4:**
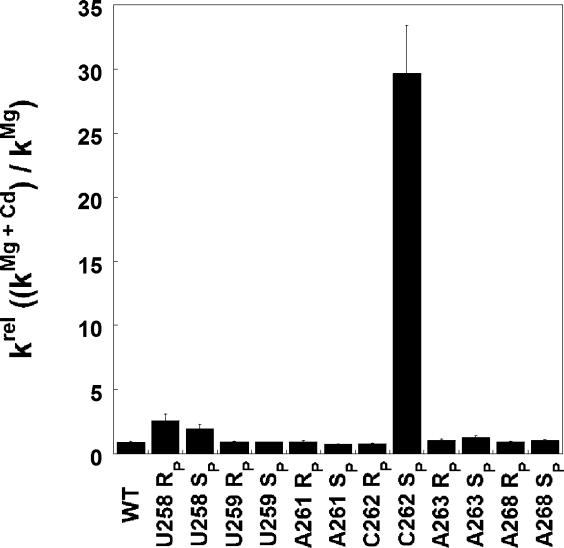
The Effect of Cd^2+^ on Activity of Phosphorothioate-Containing Ribozymes Reactions contained saturating G and ribozyme, and monitored cleavage of −1d,rSA_5_ (see Table 1) in the presence or absence of 0.1 mM Cd^2+^ (10 mM Mg^2+^ background). The largest Cd^2+^ stimulation occurred with the C262-*S*_P_ variant ribozyme. Significant Cd^2+^ stimulation remained unique to this ribozyme at all Cd^2+^ concentrations tested (0.1–10 mM; data not shown)**.** Concentrations of Cd^2+^ above 1 mM inhibit the ribozyme reaction with a steep concentration dependence (see, e.g., Figures S4 and S5A). The small Cd^2+^ stimulation observed for the U258 variant ribozymes is consistent with coordination of a metal ion important for structural stability, as proposed by Lindqvist et al. [78].

We first tested whether Cd^2+^, a thiophilic metal ion that can adopt octahedral coordination geometry like Mg^2+^ [60–62], stimulates the ability of the phosphorothioate containing ribozymes to catalyze oligonucleotide substrate cleavage (Figure 4). Under conditions of saturating ribozyme and G (10 mM MgCl_2_), several of the phosphorothioates affected catalysis significantly (data not shown, and see Table 2 below), but upon addition of 0.1–1.0 mM Cd^2+^, only one of the variant ribozymes, the C262-*S*_P_ variant, experienced significant stimulation (Figure 4 and data not shown). This stimulation suggested that a functionally important Cd^2+^ phosphorothioate interaction may occur at the C262-*S*_P_ position, leading us to focus predominantly on this variant ribozyme. The other positions tested, in principle, remain viable ligand candidates as the absence of rescue cannot be taken as evidence that the modification site does not serve as a metal ion ligand. However, metal ion rescue experiments analogous to those described below for the C262-*S*_P_ ribozyme suggest that none of the other phosphorothioate substitutions have large effects on binding of the known catalytic metal ions (see Figure S1).

**Table 2:**
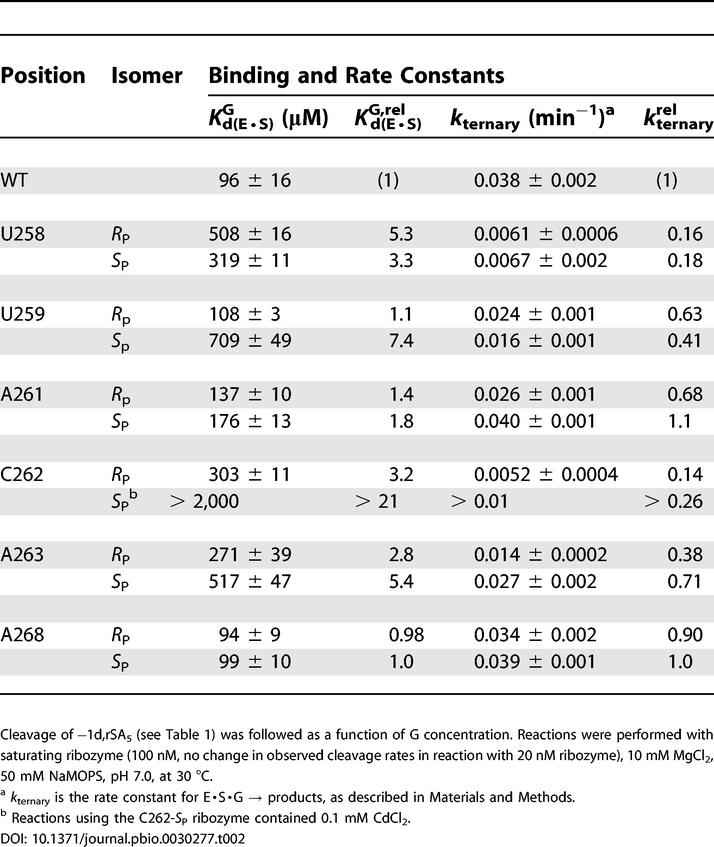
G Binding and Reactivity for Ribozymes with Site-Specific Phosphorothioate Substitutions within J6/7 and P7

### Cd^2+^ Accelerates a Non-Chemical Step in the C262-*S*_P_ Ribozyme Reaction

Metal ion rescue experiments provide definitive information about transition-state metal ion-ligand interactions only when conducted under conditions in which the same reaction steps are monitored and the chemical step limits the reaction rate [35]. To learn more about the apparent Cd^2+^ stimulation and to establish appropriate reaction conditions under which the chemical step could be monitored, we undertook basic characterization of the C262-*S*_P_ ribozyme. We first present data concerning the rate-limiting step of the C262-*S*_P_ ribozyme reaction, and then assess the effects of this mutation on individual reaction steps.

**Figure 5:**
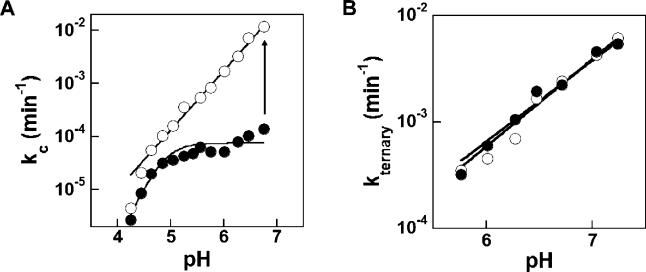
The pH Dependence of C262-*S*_P_ Ribozyme Activity Reveals a Non-Chemical Rate-Limiting Step (A) Cleavage of −1d,rSA_5_ under saturating conditions, as given in Figure 3. In the absence of Cd^2+^ (·), the pH dependence flattens above pH ∼4.7; the data are fit to *k*_max_ = 1/(1+10^n(pKa-pH)^). Addition of 1 mM Cd^2+^ (s○) leads to log-linear pH dependence (slope = 1.1 ± 0.1) over the pH range of 4–7, consistent with the chemical step being rate-limiting [33,63–65]. The Cd^2+^ rescue observed in Figure 3 results from acceleration of the non-chemical step, as indicated by the vertical arrow, such that this non-chemical step is no longer rate-limiting. The increased slope observed at low pH for reactions both with and without Cd^2+^ is consistent with ribozyme inhibition due to multiple independent protonations [65]. (B) Cleavage of −1r,dSA_5_ under saturating ribozyme and G conditions. Cleavage of −1r,dSA_5_ is log-linearly dependent on pH with and without added Cd^2+^ (0 mM Cd^2+^, • ; 1 mM Cd^2+^, s○) (best fit slopes of 0.8 in both cases), consistent with the chemical step being rate-limiting in both cases.

Previous work established that, in the wild-type (WT) ribozyme, the chemical step follows a pre-equilibrium loss of a proton, presumably from the 3′-hydroxyl of the attacking G (see Figure 1). This leads to a log-linear pH dependence with a slope of one under conditions of rate-limiting chemistry [33,63–65]. However, in the absence of Cd^2+^, the pH dependence for cleavage of the −1d,rSA_5_ substrate (Table 1) by the C262-*S*_P_ variant ribozyme is essentially flat above pH 5 (Figure 5A, closed circles), in contrast to the slope of one for the WT ribozyme [63,65]. This difference suggests that, under the reaction conditions, a conformational change limits the rate for the variant ribozyme. In the presence of low concentrations of Cd^2+^, the variant behaves more like the WT ribozyme, exhibiting a log-linear pH dependence with slope one (Figure 5A, open circles). This Cd^2+^-induced change in pH dependence suggests that Cd^2+^ accelerates the non-chemical step sufficiently to render the chemical step rate-limiting throughout the entire pH range.

Although the molecular basis for the rate-limiting, non-chemical step and its stimulation by Cd^2+^ in the C262-*S*_P_ ribozyme reaction remains undefined, comparison to the reaction of −1r,dSA_5_ offers some insight. The −1r,dSA_5_ substrate reacts from the ternary complex (E·S·G) with a log-linear pH dependence whose slope approximates one, even in the absence of Cd^2+^ (Figure 5B, closed circles). Addition of Cd^2+^ has no effect on the reaction rate or the pH-dependence (Figure 5B, open circles). These results suggest that the chemical step limits the reaction of −1r,dSA_5_ both in the presence and absence of Cd^2+^. As −1r,dSA_5_ binds primarily in the open complex with the WT ribozyme (see Figure 3) [36,66] and −1d,rSA_5_ binds in the closed complex [57,58], the rate-limiting non-chemical step observed with −1d,rSA_5_ may reflect phosphorothioate-induced formation of an altered closed complex that must rearrange prior to the chemical step. Acceleration of this non-chemical step depends on Cd^2+^; increasing the Mg^2+^ concentration does not accelerate this step, nor does Mn^2+^ or Zn^2+^ (data not shown). The Cd^2+^ dependence suggests that this non-chemical step may require metal ion coordination to the backbone phosphorothioate. Future study of this metal-ion-dependent non-chemical step revealed by phosphorothioate incorporation may provide an opportunity for deeper understanding of the role of metal ion coordination in conformational rearrangements within RNA.

This initial characterization of the C262-*S*_P_ ribozyme allowed us to choose appropriate reaction conditions for further characterization. For analyzing the binding of G and oligonucleotide substrates to the variant ribozyme, we included Cd^2+^ in sufficient but subsaturating concentrations to render the chemical step rate-limiting without influencing the measured binding constants (i.e., subsaturating Cd^2+^ in order to have no significant effect on substrate affinities; [Cd^2+^] ≤ 1 mM) [35]. For metal ion rescue reactions in which a constant Cd^2+^ concentration was not feasible, we chose oligonucleotide substrates (Table 1) or reaction pHs that render the chemical step rate-limiting.

### Phosphorothioate Substitution Effects on Reactivity and Binding

We analyzed the 12 phosphorothioate-substituted variants within the J6/7 and P7 regions of the ribozyme (see Figure 2A) under a variety of reaction conditions. As noted above, reactions with the C262-*S*_P_ variant contained Cd^2+^ to maintain rate-limiting chemistry. Cd^2+^ had no significant effect on the behavior of the other ribozymes (data not shown). Representative data from this survey—phosphorothioate substitution effects on the reaction E·S·G → products (*k*_ternary_) and G binding to the E·S complex 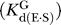
—are given in Table 2. This survey revealed one position at which phosphorothioate substitution strongly perturbs (greater than 10-fold) G binding: the *S*_P_ phosphorothioate at nucleotide C262, the same variant that exhibited Cd^2+^ stimulation.

Based on the G-binding results, the Cd^2+^ rescue of a non-chemical step, and screens for the effects of J6/7 and P7 phosphorothioates on rescue by the catalytic metal ions (see Figure S1), we decided to characterize the C262-*S*_P_ ribozyme further, focusing on how the phosphorothioate affects the individual reaction steps (see Figure 3). We found that the C262-*S*_P_ substitution had no significant effect on oligonucleotide substrate association or dissociation (Table S1). We then further investigated the observed effect of the C262-*S*_P_ phosphorothioate substitution on G binding. In reactions with the −(1–3)d,rSA_5_ substrate, which binds to the WT ribozyme in the open complex (E·S)_O_ (based on observed G-binding affinity [56,67] and Mn^2+^ stimulation of cleavage activity [66] [data not shown]), G binds to the C262-*S*_P_ variant and the WT ribozyme with the same affinity. Thus, in the absence of docked oligonucleotide substrate, the C262-*S*_P_ phosphorothioate has no effect on G binding (Table 3). The C262-*S*_P_ phosphorothioate affects subsequent reaction steps, however. The ternary open complex, (E·S·G)_O_, for the variant reacts about 40-fold slower than that for the WT ribozyme, suggesting possible effects on oligonucleotide substrate docking into the G-bound active site or on the chemical step.

**Table 3:**
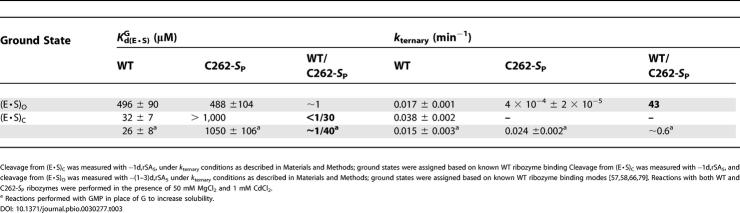
G Binding and Reactivity with WT and C262-*S*_P_ Ribozymes

In the presence of WT ribozyme, G and S bind cooperatively to give the ternary closed complex, (E·S·G)_C_. G binds to (E·S)_C_ five- to ten-fold tighter than to (E·S)_O_ or free enzyme [56,67]. Using −1d,rSA_5_ to form (E·S)_C_ and −(1–3)d,rSA_5_ to form (E·S)_O_, we reproduced this coupling for the WT ribozyme (Table 3). In contrast, the C262-*S*_P_ ribozyme exhibited no coupling. Indeed, the presence of docked oligonucleotide substrate with the C262-*S*_P_ ribozyme weakens G binding. Overall, the C262-*S*_P_ ribozyme closed complex has an approximately 40-fold weaker affinity for G than does the WT ribozyme closed complex (Table 3). Reaction chemistry from the ternary closed complex remains unaffected, as (E·S·G)_C_ formed with −1d,rSA_5_ has the same rate for the WT and C262-*S*_P_ ribozymes (Table 3).

Previous work has linked the coupled binding of G and the oligonucleotide substrate to M_C_, which mediates contacts to the G 2′-hydroxyl and the scissile phosphate *pro*-*S*_P_ oxygen (see Figure 1) [68,69]. The loss of coupled binding between the oligonucleotide substrate and G induced upon C262-*S*_P_ phosphorothioate substitution therefore raised the possibility that the C262 *pro*-*S*_P_ phosphoryl oxygen resides near the M_C_ binding site in the ribozyme tertiary structure.

### A Linkage between C262-*S*_P_ and One of the Catalytic Metal Ions

The apparent functional connection between the C262 *pro*-*S*_P_ phosphoryl oxygen atom and the metal ion bound at site C (M_C_) could occur through direct interaction of this oxygen with M_C,_ or indirectly through a chain of interactions. We used the C262-*S*_P_ ribozyme to ascertain whether direct coordination occurs, analyzing the metal ion rescue behavior for each of the previously identified metal ion sites. If the C262-*S*_P_ phosphorothioate directly coordinates to a Cd^2+^ ion bound at one of the known catalytic metal ion binding sites, we expect the stronger Cd^2+^–sulfur interaction to shift the metal ion rescue profile toward lower Cd^2+^ concentration relative to the WT ribozyme.

We determined the M_A_, M_B_, and M_C_ profiles for the WT and C262-*S*_P_ ribozymes according to approaches described previously [35,36,68]. Table 4 lists the substrates and kinetic regimes for the reactions used to obtain each rescue profile. For each rescue profile, WT and mutant ribozyme reactions with both modified and unmodified substrates must start from the same ground state and monitor the chemical step. Unless indicated otherwise, we chose substrates and conditions so that the substrate bearing the modification is not bound within the active site in the starting ground state. This ensures that substrate modifications have no effect on ground-state binding of the rescuing metal ion to the WT and mutant ribozymes. The supporting information provides full details of the methods, models, and equations used to fit the Cd^2+^ rescue dependencies.

**Table 4:**
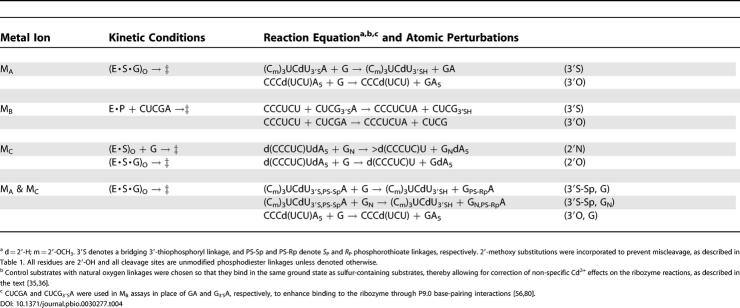
Metal Rescue Assays Used to Probe Catalytic Metal Ions in the *Tetrahymena* Ribozyme

**Figure 6:**
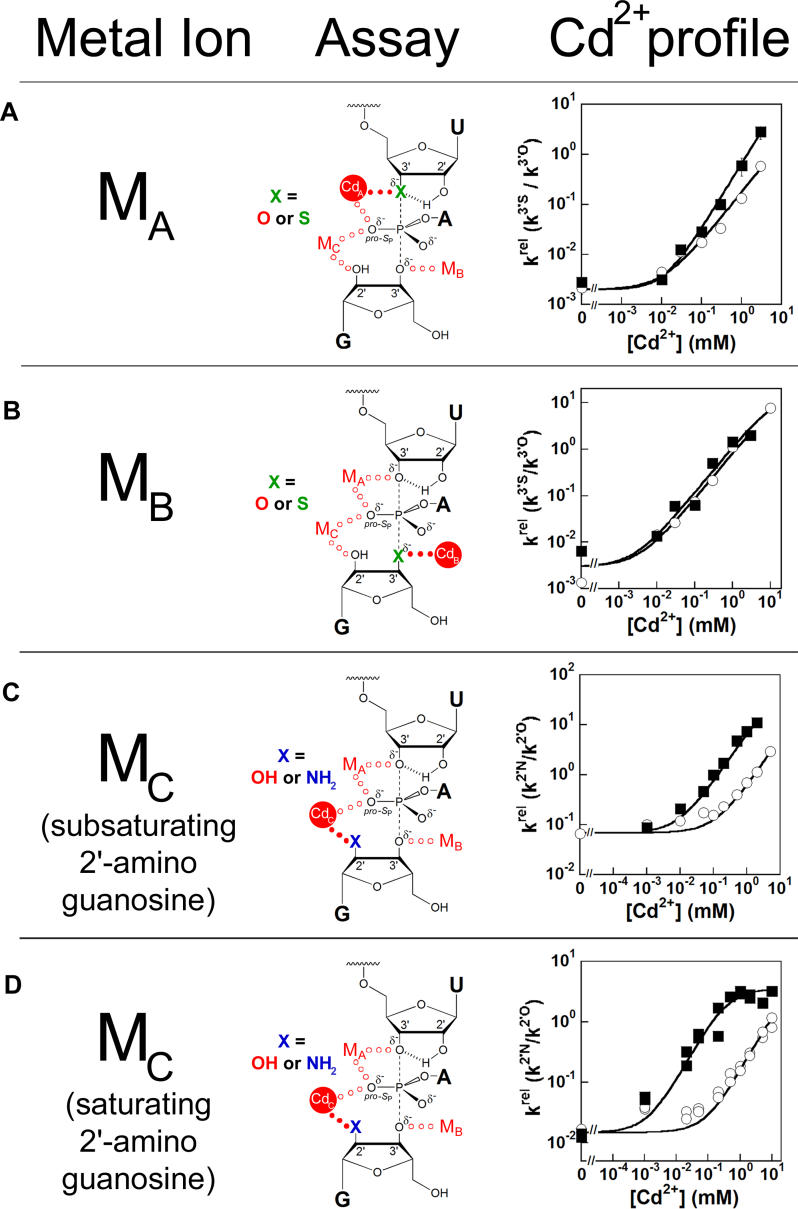
The C262-*S*_P_ Phosphorothioate Perturbs the Rescue Profile of M_C_ Cd^2+^ rescue profiles for M_A_, M_B_, and M_C_ with WT (s○) and C262-*S*_P_ (▪) ribozymes are displayed. In each case, the specific metal ion-substrate contact being probed is indicated by closed red dots. (A) M_A_ rescue of S_m3′S_ cleavage. In the *Tetrahymena* ribozyme transition-state model, the oligonucleotide substrate 3′-thiophosphoryl modification at U(-1) is shown in green. M_A_ rescue reactions monitoring cleavage of −(1–3d)rSA_5_ and S_m3′S_ were carried out under E·S·G → products (*k*_ternary_) conditions as described in Materials and Methods *.* The Cd^2+^ profiles for M_A_ rescue are fit to a Hill equation, facilitating comparison between the WT and C262-*S*_P_ ribozyme profiles (see Protocol S1). The fits give Hill constants of 1 and 1.2 for the WT and C262-*S*_P_ ribozymes, respectively. Error bars not seen are obscured by the data symbols. (B) M_B_ rescue of CUCG_3′S_A cleavage. In the transition-state model, the G 3′-thio modification is shown in green. M_B_ rescue reactions monitoring cleavage of CUCGA and CUCG_3′S_A were performed under E·P + CUCG_3′X_A → products [(*k*_c_/*K*_m_)^CUCG(3′X)A^] conditions, as described in Materials and Methods. M_B_ rescue is fit to a model in which one Cd^2+^ ion binds to E·P and stimulates reaction of CUCG_3′S_A. (C) M_C_ rescue of −1r,dSA_5_ cleavage by subsaturating G_N_ ((*k*_c_/*K*_m_)^G(or G_N_^^)^ conditions). In the transition-state model, the 2′-amino group of the G nucleophile is shown in blue. M_C_ rescue of −1r,dSA_5_ cleavage under E·S + G(or G_N_) → products ((*k*_c_/*K*_m_)^G(or G_N_^^)^ conditions) was performed as described in Materials and Methods. *k* ^rel^ data were fit to a model in which one Cd^2+^ ion binds and rescues reaction with G_N_. (D) M_C_ rescue of −1r,dSA_5_ cleavage by saturating G_N_. In the transition-state model, the 2′-amino group of the G nucleophile is again shown in blue. M_C_ rescue of −1r,dSA_5_ cleavage under E·S·G/G_N_→ products (*k*_ternary_) conditions was measured as described in Materials and Methods. *k* ^rel^ data were fit to a model in which one Cd^2+^ ion binds and rescues reaction with G_N_; with the C262-*S*_P_ ribozyme, the fit to this model gives 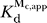 = 0.22 ± 0.08 mM. Cd^2+^ dependencies of observed cleavage rates in the M_A_, M_B_, and M_C_ rescue reactions are displayed in Figures S2, S3, and S4, respectively.

To monitor Cd^2+^ binding at the metal ion site A, we followed the reactivity of an oligonucleotide substrate containing a 3′-thiophosphoryl linkage at the cleavage site, S_m3′S_ (Figure 6A) [21,35]; i.e., Cd^2+^ specifically rescues the cleavage rate of S_m3′S_ relative to the unmodified 3′-oxygen oligonucleotide substrate (*k* ^rel^, *k* ^3′S^/*k* ^3′O^). The C262-*S*_P_ and WT ribozymes exhibit nearly identical profiles for rescue at metal ion site A (Figure 6A). The small deviation at high Cd^2+^ is beyond experimental error (as indicated by error bars in Figure 6A) and may reflect an indirect effect from Cd^2+^ occupancy at a different site (see Protocol S1 ). The lack of significant change in Cd^2+^ rescue at metal site A provides no evidence for direct contact between the *S*_P_-phosphorothioate at residue 262 and a Cd^2+^ ion binding at metal site A. Therefore, we conclude that the C262 *pro-S*_P_ phosphoryl oxygen in the WT ribozyme is not a ligand for M_A_.

To follow Cd^2+^ binding to the M_B_ site, we monitored Cd^2+^ rescue of the reactivity of CUCG_3′S_A relative to CUCGA in the reverse reaction with E·P (Equation 2) [20,35]. CUCG_3′S_A contains a 3′-thiophosphoryl linkage at the cleavage site.



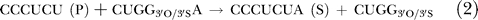



In reactions catalyzed by both the WT and C262-*S*_P_ ribozymes, Cd^2+^ specifically stimulates the reactivity of CUCG_3′S_A more than 500-fold. This rescue fits well to a model in which a single Cd^2+^ ion binds to the M_B_ site and rescues the reaction (Figure 6B). As C262-*S*_P_ phosphorothioate incorporation exhibits no effect on the M_B_ Cd^2+^ rescue profile, we conclude that the C262 *pro*-*S*_P_ phosphoryl oxygen in the WT ribozyme is not a ligand for M_B_.

To follow Cd^2+^ binding to the M_C_ site, we monitored Cd^2+^ rescue of oligonucleotide substrate cleavage by 2′-aminoguanosine (G_N_) relative to G [19,35,68]. We first probed Cd^2+^ binding to the M_C_ site in the E·S complex, conducting reactions at subsaturating G or G_N_ concentrations ((*k*_c_/*K*_m_)^G^ or (*k*_c_/*K*_m_ 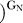 conditions). Specific Cd^2+^ stimulation of cleavage with G_N_ occurs with both the WT and C262-*S*_P_ ribozymes, giving linear rescue curves throughout the Cd^2+^ concentration range tested with slopes that reflect a single Cd^2+^ stimulating cleavage by G_N_ (Figure 6C). In contrast to the M_A_ and M_B_ rescue profiles discussed above, the M_C_ rescue curve for the C262-*S*_P_ variant shifts to the left, with 16-fold lower Cd^2+^ concentrations required to achieve the same level of rescue as the WT. This shift suggests that the *S*_P_-phosphorothioate at nucleotide C262 interacts directly with the Cd^2+^ ion binding at the M_C_ site, a model we test further below.

The M_C_ rescue profiles for both the WT and C262-*S*_P_ ribozymes increase linearly up to the highest experimentally accessible Cd^2+^ concentrations (Figure 6C), indicating that the rescuing Cd^2+^ ion binds to the E·S complexes of these ribozymes under these conditions with a dissociation constant that exceeds 10 mM. Without saturation behavior, we cannot definitively ascertain whether the C262 phosphorothioate-induced shift in the M_C_ rescue profile emanates from tighter Cd^2+^ binding. Consequently, we probed Cd^2+^ binding to the M_C_ site in the presence of saturating G_N_. Bound G_N_ provides M_C_ with an additional ligand, the nitrogen of the 2′-amino group, which should interact strongly with Cd^2+^ [70].

We conducted the rescue experiment as described above but included G_N_ at saturating concentration to form the E·S·G_N_ ternary complex. As with subsaturating G_N_, both the WT and C262-*S*_P_ ribozymes experienced significant specific Cd^2+^ rescue (Figure 6D). The rescue profile for the WT E·S·G_N_ complex remains linear throughout the Cd^2+^ concentration range tested, indicating that even in the presence of bound G_N_, Cd^2+^ binds to the M_C_ site with an apparent affinity of greater than 10 mM (50 mM Mg^2+^ background). In striking contrast, the Cd^2+^ rescue profile for reaction of the C262-*S*_P_ E·S·G_N_ complex exhibits saturation behavior, with an apparent dissociation constant of 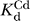 ∼ 0.2 mM. The C262-*S*_P_ E·S·G_N_ ternary complex therefore binds the rescuing Cd^2+^ ion more than 50-fold tighter than does C262-*S*_P_ ribozyme E·S binary complex, suggesting that the 2′-amino group of G_N_ interacts with M_C_ in the ground state. Moreover, the C262-*S*_P_ E·S·G_N_ complex binds the rescuing Cd^2+^ ion more than 50-fold tighter than does the corresponding WT ternary complex, showing that the phosphorothioate substitution at C262 enhances Cd^2+^ binding to metal ion site C. Taken together, these data further support a model in which the C262-*S*_P_ phosphorothioate interacts directly with the Cd^2+^ ion in the M_C_ site and strongly implicate the C262 *pro-S*_P_ oxygen as a ligand for M_C_ in the WT ribozyme.

### An Independent Test of the C262 *pro-S*_P_ Phosphoryl Oxygen as a M_C_ Ligand

**Figure 7:**
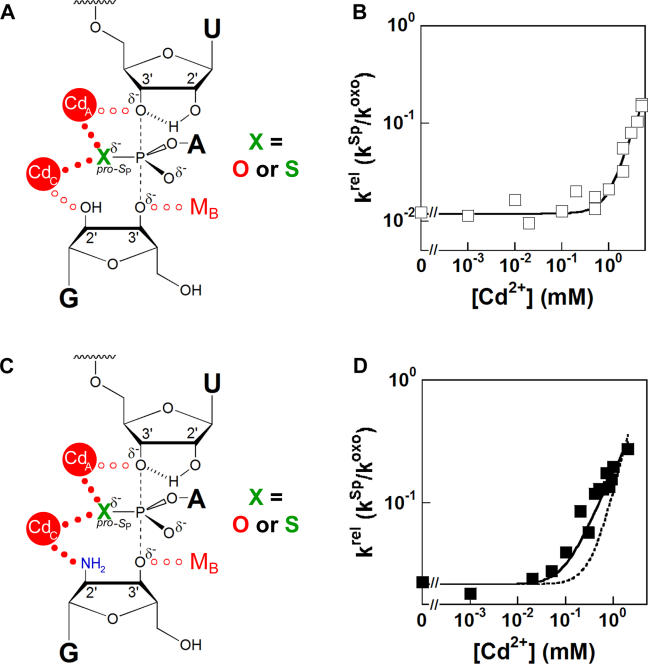
Cd^2+^ Rescue of S_Sp_ Cleavage by the C262-*S*_P_ Ribozyme Reactions were performed under E·S·G/G_N_ → products (*k*_ternary_) conditions, as described in Materials and Methods, with −(1–3)d,rSA_5_ serving as the unmodified control oligonucleotide substrate. Cd^2+^ dependencies for oligonucleotide substrate cleavage by G and G_N_ are displayed in Figure S5. (A) Model of the *Tetrahymena* ribozyme transition state for rescue of S_Sp_ reaction by Cd^2+^ ions bound at M_A_ and M_c_. Closed red circles indicate metal ion coordinations considered important for rescue, and the *S*_P_ phosphorothioate modification at the scissile phosphate is denoted in green. (B) *k* ^rel^ versus Cd^2+^ for cleavage of S_Sp_ with G. The data are fit to a model in which two Cd^2+^ ions rescue reaction, with neither Cd^2+^ binding site saturating (see Protocol S1). (C) Model of the *Tetrahymena* ribozyme transition state for rescue of S_Sp_ cleavage by two Cd^2+^ ions bound at M_A_ and M_C_ in the presence of saturating G_N_. Metal ion coordinations important for rescue are denoted by closed red circles, and the G_N_ amino group is shown in blue. (D) *k* ^rel^ versus Cd^2+^ for cleavage of S_Sp_ with G_N_. The dotted line is a fit to the model used in (B), for two Cd^2+^ ions rescuing reaction and neither Cd^2+^ binding site saturating. The solid line is a fit to a model in which two Cd^2+^ ions rescue reaction, with one site saturating with a *K* ^M_c_^_d,app_ = 0.2 mM (see Protocol S1).

The reaction of an oligonucleotide substrate bearing a *S*_P_-phosphorothioate at the cleavage site (S_Sp_, Table 1) also experiences Cd^2+^ stimulation with the WT ribozyme. The Cd^2+^ rescue profile for this modified oligonucleotide substrate exhibits a slope of two, suggesting that two Cd^2+^ ions contact the non-bridging sulfur atom in the transition state [36]. Thermodynamic fingerprint analysis established that these rescuing metal ions bind at sites A and C (Figure 7A) [36]. The S_Sp_ oligonucleotide substrate therefore offers another strategy by which to test whether the C262-*S*_P_ phosphorothioate enhances Cd^2+^ binding to the M_C_ site. If the M_C_ site in the C262-*S*_P_ ribozyme becomes saturable in the presence of bound G_N_, as described in the previous section, then the rescue profile for S_Sp_ cleavage should transition from an apparent dependence on two Cd^2+^ ions to a dependence on one Cd^2+^ ion. The results described below meet this prediction, thereby providing additional quantitative support for the assignment of the *pro-S*_P_ phosphoryl atom of residue C262 as a ligand for M_C_.

The Cd^2+^ rescue profile for S_Sp_ cleavage by C262-*S*_P_ ribozyme with saturating G fits well to a model with two Cd^2+^ ions rescuing S_Sp_ cleavage with neither metal ion saturating, analogous to that for the WT ribozyme (Figure 7B) [36]. This profile matches expected rescue behavior, given that neither the M_A_ nor M_C_ (subsaturating G_N_) individual rescue profiles exhibit saturation with the C262-*S*_P_ ribozyme (see Figures 6A and 6C, respectively). In contrast, with saturating G_N_, Cd^2+^ rescue of S_Sp_ cleavage by the C262-*S*_P_ ribozyme no longer fits well to a dependence on two nonsaturating Cd^2+^ ions (see Figure 7C and the dashed line in Figure 7D). Rather, the best fit gives a dependence on only one Cd^2+^ ion, suggesting that one of the two metal ion sites involved in rescuing the S_Sp_ reaction saturates. Assuming that one Cd^2+^ ion binds to the C262-*S*_P_ ribozyme with 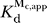 = 0.2 mM, the value determined for Cd^2+^ binding to metal site C in the presence of saturating G_N_ (see Figure 6D), we obtain the solid line in Figure 7D. In contrast, for the WT ribozyme, Cd^2+^ rescue of the S_Sp_ reaction in the presence of saturating G_N_ exhibits a dependence on two Cd^2+^ ions up to the highest accessible Cd^2+^ concentration [36]. Therefore, as predicted, the C262-*S*_P_ phosphorothioate substitution enhances binding of one of the rescuing Cd^2+^ ions in the reaction of S_Sp_, strongly supporting the conclusion that M_C_ interacts directly with the *pro-S*_P_ atom of the C262 phosphate.

## Discussion

**Figure 8:**
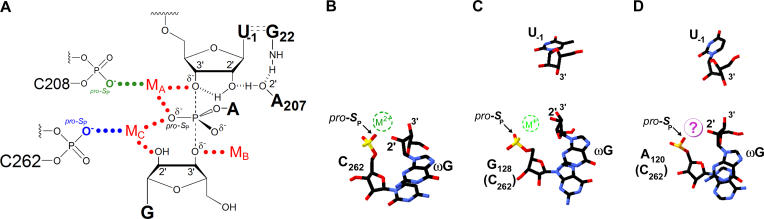
Functional and Structural Models of Group I Intron Active Sites (A) Model of the *Tetrahymena* ribozyme transition state from functional data with the C262 *pro*-*S*_P_ phosphoryl oxygen and C208 *pro*-*S*_P_ oxygen coordinating to M_C_ and M_A_, respectively ([35,36,53,58,71,72, 74], data herein, and AVK, JLH, JAP, and DH, unpublished results). (B) Model of Mg^2+^ binding in the crystal structure of a thermostable variant of the *Tetrahymena* group I ribozyme (derived from PDB file 1X8W) [14]. The model shown is derived from molecule C, although the position of the metal ion appears to vary among the four molecules observed in the asymmetric unit. The putative Mg^2+^ ion (dashed green circle) is 2.4 Å from the *pro-S*_P_ oxygen of C262 and 2.1 Å from the terminal G (ωG) 2′-OH. U(-1) is not shown, as the crystallized form of the thermostable *Tetrahymena* variant ribozyme lacks this nucleotide. (C) Model of K^+^ binding from the crystal structure of the *Azoarcus* group I ribozyme (derived from PDB file 1T42) [15]. The putative K^+^ ion (dashed green circle) is 2.4 Å from the *pro-S*_P_ oxygen of G128 (C262 homologue) and 2.8 Å from the modeled position for the terminal G (ωG) 2′-OH; the *Azoarcus* intron construct that was crystallized contained 2′-deoxyguanosine at ωG, and electron density for the K^+^ ion is observed only in the presence of this 2′-deoxyguanosine modification at ωG. (D) Model of proposed Mg^2+^ binding site derived from the crystal structure of the Twort group I ribozyme [13]. The crystallographic data lack clear density for a metal ion in the region expected for the M_C_ binding site but show the *pro*-*S*_P_ phosphoryl oxygen of A120 (C262 homologue) and the 2′-OH of ωG appropriately juxtaposed to coordinate a single Mg^2+^ ion (purple circle).

Metalloenzymes that catalyze phosphoryl transfer play multiple roles throughout biology, serving as kinases, phosphatases, polymerases, and nucleases, among other functions [1–10]. We have established a powerful new experimental paradigm with which to identify ligands that coordinate to catalytic metal ions. Our analysis of simultaneous atomic perturbations within the *Tetrahymena* ribozyme core and its substrates, under conditions that allow valid thermodynamic comparisons, provides strong evidence that the *pro*-*S*_P_ oxygen at residue C262 serves as a ligand for metal ion C (M_C_, Figure 8A). A single phosphorothioate substitution at this site, alone and in combination with G_N_, changes the metal ion affinity and specificity at the M_C_ site while having little or no effect on the M_A_ and M_B_ sites. Although we cannot determine unambiguously whether the interaction between the *pro*-*S*_P_ oxygen at C262 and M_C_ occurs via outer sphere or inner sphere coordination in the natural ribozyme, the presence of a direct, inner sphere metal ion-ligand interaction provides the simplest model to account for our observations. More extensive rearrangement by the ribozyme active site to accommodate the backbone mutation appears less likely, as the metal ion that contacts C262-*S*_P_ satisfies all the transition-state contacts proposed for M_C_. Further demonstrating the efficacy of this approach, detailed analysis of phosphorothioate mutations in the P4 and J5/4 regions of the ribozyme core identified the *pro-S*_P_ oxygen at position C208 as a ligand for M_A_ (AVK, JLH, JAP, and DH, unpublished data), consistent with the previous proposal of Szewczak et al. [53].

Under some conditions, the C262-*S*_P_ ribozyme E·S·G ternary complex must undergo a rate-limiting conformational change en route to the chemical transition state. Cd^2+^ accelerates this non-chemical step in this C262-*S*_P_ ribozyme, suggesting that M_C_, which interacts with the phosphorothioate during the reaction, mediates this conformational change. Currently, we lack sufficient information to speculate further on the molecular basis of this conformational change. The ability to induce a new or existing conformational change may provide a future opportunity to investigate the relationship between RNA function and dynamics.

Crystal structures of three different group I introns have emerged in the past several months, providing a structural context for these functionally identified catalytic metal ligands [13–15]. The three structures converge beautifully with respect to the global architecture and reveal the highly electrostatic character of the active site, consistent with functional studies. These structures also underscore the difficulty of defining catalytic metal ion interactions, exhibiting limited agreement regarding the active-site conformation and the number, charge, and location of bound metal ions. This variability in metal ion number and location notwithstanding, the C262 *pro-S*_P_ oxygen (or its equivalent) lies in proximity to the ωG 2′-hydroxyl in all three structures (Figure 8). The *Azoarcus* and *Tetrahymena* crystals contain electron density for a metal ion within coordination distance of this phosphoryl oxygen [14,15], whereas the Twort ribozyme structure lacks such electron density [13]. The data presented herein establish the importance of this proximity for function and suggest that the active-site configuration in the crystals bears at least some relevance to the active-site configuration in the transition state. This agreement between structural and functional data for metal ion C supports the assignment of a conserved catalytic metal ion binding site at the top of the P7 helix in group I introns (see Figure 2A).

Extensive group I intron biochemical investigations, conducted for two decades in the absence of atomic resolution structures, have revealed an intricate network of interactions surrounding the reaction center in the transition state interconnected by hydrogen bonds, metal ion coordinations, and the atomic configuration of the reactants (see Figure 1). The results suggest a constellation of three metal ions at the active site [35,36], making five atomic interactions to the transition state. In addition to these catalytic metal ion coordinations, the 2′-hydroxyl group of U(-1) donates a hydrogen bond to the adjacent 3′-oxygen leaving group in the transition state [71]. The 2′-hydroxyl group of A207 donates a hydrogen bond to the 2′-OH of U(-1) and accepts a hydrogen bond from the exocyclic amine of G22, thereby bridging the cleavage site 2′-OH and the G·U wobble pair [58,72]. The recent group I intron crystal structures, though unable to define the number, location, or catalytic interactions of metal ions unambiguously, provide an opportunity to visualize how these functionally defined networks extend deeper into the ribozyme core.

Based on the recent crystal structure of the *Azoarcus* group I intron, an alternative model for the transition state was proposed in which only two metal ions interact with the reaction center [15]. In this model, one metal ion coordinates to the 3′-oxygen leaving group of the cleavage site uridine and the *pro*-*S*_P_ oxygen of the scissile phosphate, the same interactions proposed for M_A_ in the functional model (Figure 8A). The other metal ion coordinates directly with the 2′-hydroxyl group of G as does M_C_ in the functional model, but coordinates indirectly to the *pro*-Sp oxygen of the scissile phosphate, in contrast to the direct interaction implicated by the functional data. A metal ion interaction with the 3′-oxygen of G, mediated by M_B_ in the functional model, is absent from the crystallographic model. No direct evidence for a M_B_-binding site was obtained from the X-ray structure. The resting structure of the RNA in the crystal could adopt a conformation that differs from the active structure and thereby exclude M_B_ from the crystals. Alternatively, the M_B_ rescue profile could reflect the recruitment of a thiophilic metal ion to the active site during the reaction of the sulfur-containing G analogue. As the 3′-oxygen of the G undergoes a large charge rearrangement in the transition state, we suggest that a direct metal ion interaction with the 3′-oxygen of G is likely, such that M_B_ is present in the normal reaction or metal ion C makes both 2′- and 3′-interactions with G [73]. These different proposals for specific metal ion coordination configurations during the ribozyme reaction highlight the need for further structural and functional tests and refinements of the catalytic models.

To achieve a unified description of catalytic function that integrates the biochemically defined interaction networks and the crystallographically defined three-dimensional structure, we must establish “anchor points”—functionally verifiable linkages between transition-state interactions and the enzyme's core. Our analysis establishes the C262 *pro-S*_P_ oxygen as such an anchor point. Anchor points provide critical information about the spatial arrangement of catalytic groups within the global architecture of the enzyme. Together, the transition state model, anchor points, and the recent X-ray structures establish a powerful foundation to build toward an in-depth understanding of how cooperative structure adopted by this RNA and other enzymes engenders enormous catalytic power and exquisite specificity.

## Materials and Methods

### Materials

WT *Tetrahymena* ribozyme was prepared as described previously [34]. All oligonucleotide substrates (see Table 1) were prepared and 5′-end-labeled using standard methods [34,35,54,74]. Oligonucleotides with thio substitutions were prepared by published procedures [75]. Oligonucleotides containing phosphorothioate diastereomers were separated by anion exchange HPLC [42,54]. Reverse phase HPLC of purified diastereomers, under conditions in which the *R*_P_ diastereomer elutes before the *S*_P_, allowed assignment of each diastereomer's configuration [76].

### Ribozyme preparation

Variant ribozymes were constructed semisynthetically using successive splint-mediated ligations [77]. Synthetic oligonucleotides corresponding to nucleotides 255–274 of the ribozyme, each containing a single phosphorothioate modification at the desired mutation site, were purchased from Dharmacon (Lafayette, Colorado, United States). Following phosphorothioate separation, synthetic oligonucleotides were 5′-phosphorylated with T4 polynucleotide kinase and cold ATP. Constructs corresponding to nucleotides 22–254 and 274–409 of the *Tetrahymena* ribozyme were transcribed using DNA templates produced by PCR truncation of the plasmid-encoded ribozyme sequence, with excess GMP present in the transcription of the 3′-construct to yield a 5′-monophosphate. The transcripts were ligated to the synthetic oligonucleotide via two successive splint-mediated ligations with T4 DNA ligase to yield full-length ribozyme containing a single *R*_P_- or *S*_P_-phosphorothioate mutation at the desired site.

Ligated ribozymes appear to contain approximately 40% inactive enzyme fraction, as indicated by biphasic kinetics under conditions in which oligonucleotide substrate cleavage occurs faster than oligonucleotide substrate dissociation (data not shown). Evidence suggests that the inactive ribozyme fraction in these semisynthetic ribozymes may be due to errors at the ligation junctions resulting from ligation of a subset of transcribed RNA constructs with incorrect termini (K. Travers, V. Diankov, and DH, unpublished data). Under conditions used in this work to characterize variant ribozyme reactivity, the inactive fraction did not contribute to the cleavage activity monitored in our assays of ribozyme activity. The inactive fraction did not affect association and dissociation rate constants measured by pulse chase experiments, as evidenced by monophasic binding behavior (data not shown). The activity of WT ribozyme constructed by ligation varied less than 2-fold from that of transcribed WT ribozyme (Table S2) after accounting for the presence of the inactive ribozyme fraction. The inactive ribozyme fraction does not affect the conclusions in this work.

### General kinetic methods

All cleavage reactions were single turnover, with ribozyme in excess of radiolabeled S (S*), and were carried out at 30 °C in 50 mM buffer and 50 mM MgCl_2_ unless noted otherwise. All reactions without Cd^2+^ contained 0.1 mM EDTA. The buffers used were NaOAC (pH 4.0–5.2), NaMES (pH 5.6–6.7), and NaMOPS (pH 7.0–7.5). Reaction mixtures containing all components except Cd^2+^, EDTA, and radiolabeled oligonucleotide substrate were pre-incubated at 50 °C for 30 min to renature the ribozyme. Reactions were followed and analyzed as described previously [33,36,74].

### Determination of rate and equilibrium constants

For oligonucleotide substrates that bind to the WT ribozyme in the closed complex, e.g. −1d,rSA_5_ (see Table 1), we define *k*_c_ as the first-order rate constant for the reaction of the ternary complex (E·S·G)_C_ → products. Values of *k*_c_ were determined at pH 7.0, with ribozyme saturating with respect to oligonucleotide substrate (20–100 nM E, 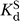 < 1 nM) and with saturating G (2 mM, 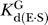 as reported in Table 2).

For cleavage of oligonucleotide substrates that are known to bind in the open complex to the WT ribozyme, e.g., −1r,dSA_5_ and S_Sp_ [36,66], we define *k*_ternary_ as the first-order rate constant for the reaction E·S·G/G_N_ → products. We also used *k*_ternary_ to describe cleavage reactions catalyzed by variant ribozymes for which the oligonucleotide substrate binding mode has not been assigned definitively. Values of *k*_ternary_ were determined at pH 7.0, with ribozyme saturating with respect to oligonucleotide substrate (20–50 nM E, ribozyme concentration at least 4-fold above , 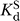 data not shown) and with saturating G or G_N_ (2 mM, 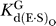 ∼ 500 μM; 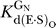 ∼ 100–150 μM) ([68], Tables 2 and 3, and data not shown).

(*k*_c_/*K*_m_)^G^ and (*k*_c_/*K*_m_ 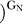 are the second-order rate constants for the reaction E·S + G (or G_N_) → products. In these experiments, we used the −1r,dSA_5_ oligonucleotide substrate that binds to the WT and variant ribozymes in the open complex [36,66]. Values of (*k*_c_/*K*_m_)^G^ and (*k*_c_/*K*_m_ 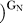 were determined at pH 7.0, with ribozyme saturating with respect to oligonucleotide substrate (20–50 nM E, ribozyme concentration at least 4-fold above , 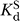
data not shown) and with subsaturating G or G_N_ (30 μM G or G_N_, 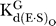 ∼ 500 μM; 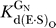 ∼ 100–150 μM) ([68], Table 3, and data not shown).

(*k*_c_/*K*_m_)^CUCG(3′X)A^ (where X = O or S) is the second-order rate constant for the reaction E·P + CUCGA → products. Values of (*k*_c_/*K*_m_)^CUCG(3′X)A^ were determined at pH 6.5 with trace amounts of radiolabeled CUCG_3′X_A and E·P subsaturating with respect to CUCG_3′X_A (X = O, 10–20 nM E·P, 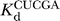 > 100 nM; X = S, 100–200 nM E·P, 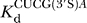 > 400 nM) (data not shown). To maintain the chemical step as rate-limiting in reactions catalyzed by the WT ribozyme, an oligonucleotide product with a 2′-deoxyribothymidine at the 3′-terminus was used (CCCUCdT); 2′-deoxyribose incorporation at this site slows the chemical step approximately 10^3^-fold [74]. Reactions in the presence of the C262-*S*_P_ variant ribozyme used an all-ribose oligonucleotide product (CCCUCU).

Association (*k*_on_) and dissociation (*k*_off_) rate constants were measured by a gel mobility shift assay using pulse-chase methods [33,56]. Specific conditions and experimental details for these experiments are described in Protocol S2.

### Experimental errors

All titrations, binding constants, and rate determinations were repeated at least three times. Error bars, when present, indicate standard deviation from at least three determinations. Error bars may be obscured by data symbols. Plots without error bars display representative data. Reported errors in all tables, both in the text and Supporting Information, are the standard deviation of at least three independent measurements.

### Data analysis

The Cd^2+^ concentration dependencies for rescue of reaction of modified substrates were analyzed according to previously described methods ([35,36] and Protocol S1).

## Supporting Information

Figure S1Phosphorothioate Effects on M_A_, M_B_, and M_C_ Rescue of Modified Substrates by Cd^2+^
Stimulation of modified ribozyme substrates was assessed by comparing cleavage activity in 50 mM Mg^2+^ alone to observed activity in the presence of 1 mM Cd^2+^ and 50 mM Mg^2+^. The cleavage rate in the presence of Cd^2+^ was divided by the observed cleavage rate in Mg^2+^ alone, resulting in a relative rate (*k* ^rel^). These *k* ^rel^ values were then normalized to the WT ribozyme; i.e., 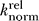 for the WT ribozyme equals one.
(A) Normalized stimulation of cleavage of an oligonucleotide substrate containing a 3′-thiophosphoryl linkage by 1 mM Cd^2+^; reactions performed as described in Figures 6 and S2 and Table 4.(B) Normalized stimulation of cleavage of a 3′-splice site analogue containing a 3′-thiophosphoryl linkage by 1 mM Cd^2+^; reactions performed as described in Figures 6 and S3 and Table 4.(C) Normalized stimulation of oligonucleotide substrate cleavage by subsaturating G_N_ by 1 mM Cd^2+^; reactions performed as described in Figures 6 and S4 and Table 4.(2.4 MB PDF).

Figure S2 Cd^2+^ Specifically Stimulates Cleavage of an Oligonucleotide Substrate Containing a 3′-Thiophosphoryl Linkage (A) [Cd^2+^] dependencies for the reaction E·S·G → products (*k*_ternary_) for −(1–3)d,rSA_5_ (s○) and S_m3′S_ (•) with the WT ribozyme (see Materials and Methods).
(B) [Cd^2+^] dependencies of the rate of reaction E·S·G → products (*k*_ternary_) for −(1–3)d,rSA_5_ (□) and S_m3′S_ (▪) with the C262-*S*_P_ ribozyme (see Materials and Methods). Error bars not seen are obscured by data symbols.
(2.8 MB PDF).

Figure S3 Cd^2+^ Specifically Stimulates Cleavage of a 3′-Splice Site Analogue Containing a 3′-Thiophosphoryl Linkage (A) [Cd^2+^] dependencies of the rate of the reaction E·P + CUCG_3′X_A → products ((*k*_c_/*K*_m_)^CUCG(3′X)A^) for CUCGA (s○) and CUCG_3′S_A (•) with the WT ribozyme (see Materials and Methods)
(B) [Cd^2+^] dependencies of the rate of the reaction E·P + CUCG_3′X_A → products ((*k*_c_/*K*_m_)^CUCG(3′X)A^) for CUCGA (□) and CUCG_3′S_A (▪) with the C262-*S*_P_ ribozyme (see Materials and Methods). Error bars not seen are obscured by data symbols.
(3.1 MB PDF).

Figure S4 Cd^2+^ Specifically Stimulates Oligonucleotide Substrate Cleavage by G_N_
(A) [Cd^2+^] dependencies of the rate of the reaction E·S + G (or G_N_) → products ((*k*_c_/*K*_m_)^G(or GN)^) for G(s○) and G_N_(•) cleavage of −1r,dSA_5_ with the WT ribozyme (see Materials and Methods).
(B) [Cd^2+^] dependencies of the rate of the reaction E·S + G (or G_N_) → products ((*k*_c_/*K*_m_)^G(or GN)^) for G (□) and G_N_(▪) cleavage of −1r,dSA_5_ with the C262-*S*_P_ ribozyme (see Materials and Methods).
(C) [Cd^2+^] dependencies of the rate of the reaction E·S·G/G_N_ → products (*k*_ternary_) for G(s○) and G_N_(•) cleavage of −1r,dSA_5_ with the WT ribozyme (see Materials and Methods).
D) [Cd^2+^] dependencies of the rate of the reaction E·S·G/G_N_ → products (*k*_ternary_) for G(□) and G_N_(▪) cleavage of −1r,dSA_5_ with the C262-*S*_P_ ribozyme (see Materials and Methods).
(2.9 MB PDF).

Figure S5 Cd^2+^ Specifically Stimulates Cleavage of an Oligonucleotide Substrate Containing a *S*_P_-Phosphorothioate Substitution at the Cleavage Site (A) [Cd^2+^] dependencies of the rate of the reaction E·S·G → products [*k*_ternary_] for S_Sp_ (▪) and −(1–3)d,rSA_5_ (□) cleavage with G with the C262-*S*_P_ ribozyme (see Materials and Methods).
(B) [Cd^2+^] dependencies of the rate of the reaction E·S·G/G_N_ → products [*k*_ternary_] for S_Sp_ cleavage by GN (▪) and −(1–3)d,rSA_5_ cleavage by G (□) with the C262-*S*_P_ ribozyme (see Materials and Methods).
(2.8 MB PDF).

Protocol S1 Analysis of Cd^2+^ Rescue Profiles (126 KB DOC).

Protocol S2 Measuring Oligonucleotide Substrate Association and Dissociation Rate Constants (26 KB DOC).

Table S1 Oligonucleotide Substrate Binding to WT and C262-*S*_P_ Ribozymes (25 KB DOC).

Table S2 G Binding and Reactivity of WT Ribozymes Constructed by Transcription and Ligation (19 KB DOC).

### Accession Numbers

The Protein Data Bank (http://www.rcsb.org/pdb/) accession numbers for the group I intron crystal structures discussed in this paper are *Azoarcus* (PDB ID 1U6B), *Tetrahymena* (PDB ID 1X8W), and Twort ribozyme (PDB ID 1Y0Q).

## Abbreviations

E: ribozyme
G: guanosine
G_N_: 2′-aminoguanosine
S: oligonucleotide substrate
WT: wild-type
