# Supplementary material for: Functional Identification of Catalytic Metal Ion Binding Sites within RNA
Source: PLoS Biol. 2005 Aug 16;3(9):e277. doi: 10.1371/journal.pbio.0030277 (PMC1184590; doi:10.1371/journal.pbio.0030277)
Supplement: Supplementary file 1 [file pbio.0030277.sg001.pdf]

Figure S1

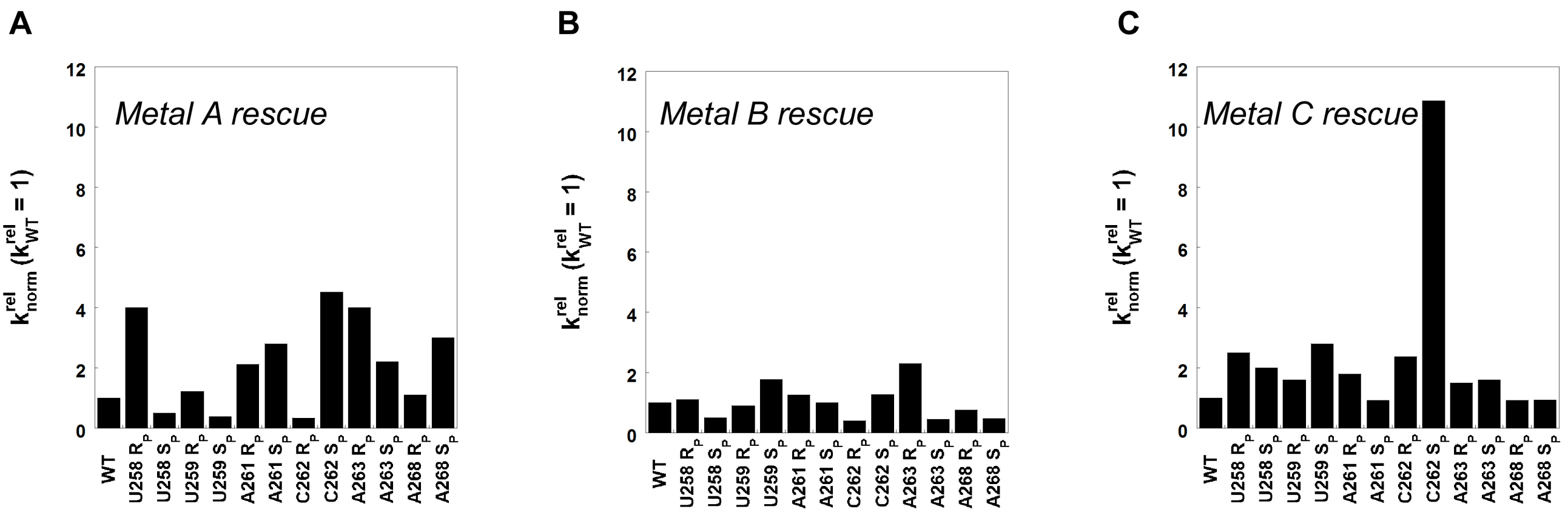
A
B
C
Metal A rescue
Metal B rescue
Metal C rescue
k^{rel}_{norm} (k^{rel}_{WT} = 1)
0
2
4
6
8
10
12
WT
U258 R_P
U258 S_P
U259 R_P
U259 S_P
A261 R_P
A261 S_P
C262 R_P
C262 S_P
A263 R_P
A263 S_P
A268 R_P
A268 S_P
