# Supplementary material for: Functional Identification of Catalytic Metal Ion Binding Sites within RNA
Source: PLoS Biol. 2005 Aug 16;3(9):e277. doi: 10.1371/journal.pbio.0030277 (PMC1184590; doi:10.1371/journal.pbio.0030277)
Supplement: Supplementary file 3 [file pbio.0030277.sg003.pdf]

Figure S3

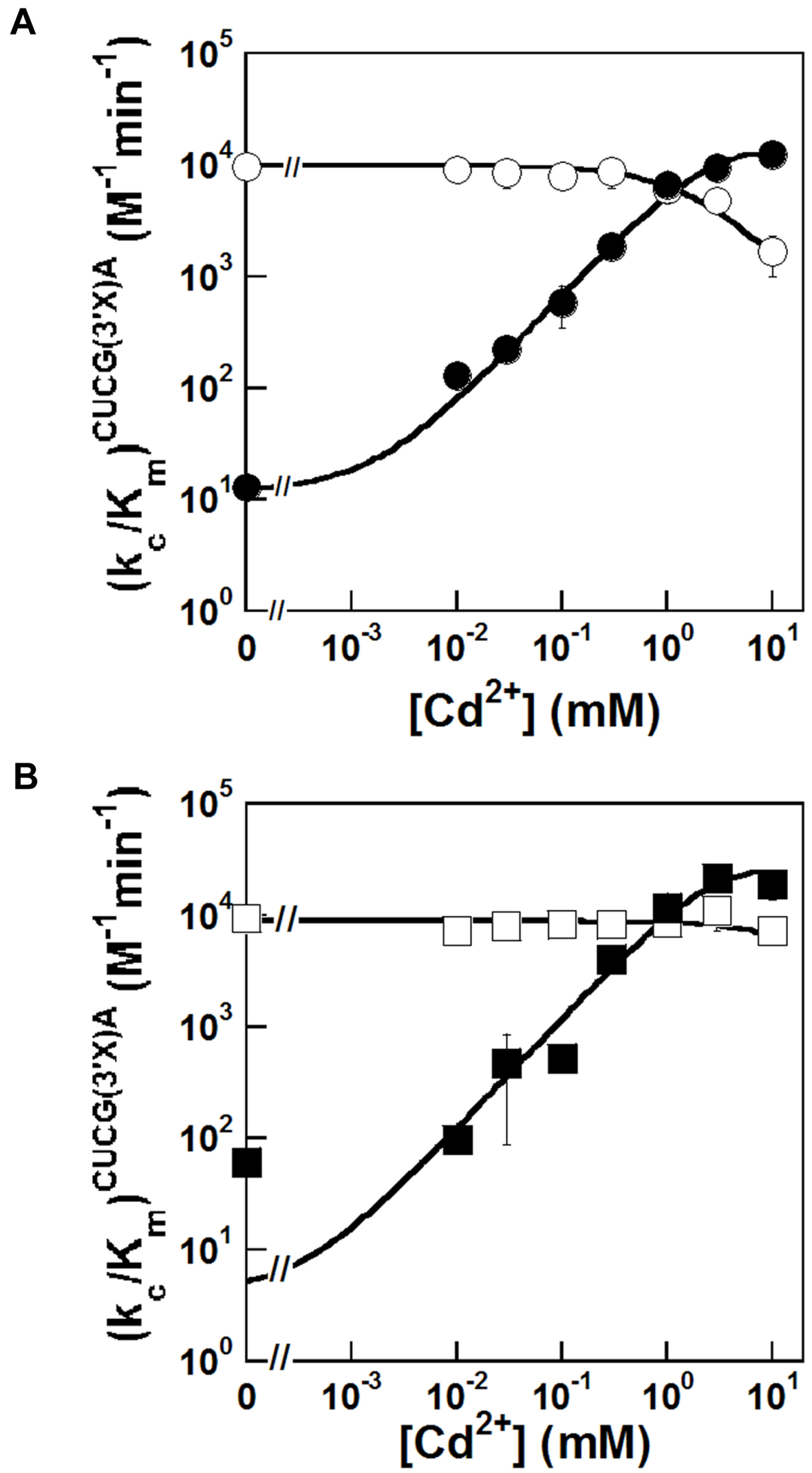
A
$(k_c/K_m)^{CUCG(3'X)A}$ $(M^{-1}min^{-1})$
10^5
10^4
10^3
10^2
10^1
10^0
0
10^{-3}
10^{-2}
10^{-1}
10^0
10^1
$[Cd^{2+}]$ (mM)
B
$(k_c/K_m)^{CUCG(3'X)A}$ $(M^{-1}min^{-1})$
10^5
10^4
10^3
10^2
10^1
10^0
0
10^{-3}
10^{-2}
10^{-1}
10^0
10^1
$[Cd^{2+}]$ (mM)
