# Supplementary material for: Functional Identification of Catalytic Metal Ion Binding Sites within RNA
Source: PLoS Biol. 2005 Aug 16;3(9):e277. doi: 10.1371/journal.pbio.0030277 (PMC1184590; doi:10.1371/journal.pbio.0030277)
Supplement: Supplementary file 5 [file pbio.0030277.sg005.pdf]

Figure S5

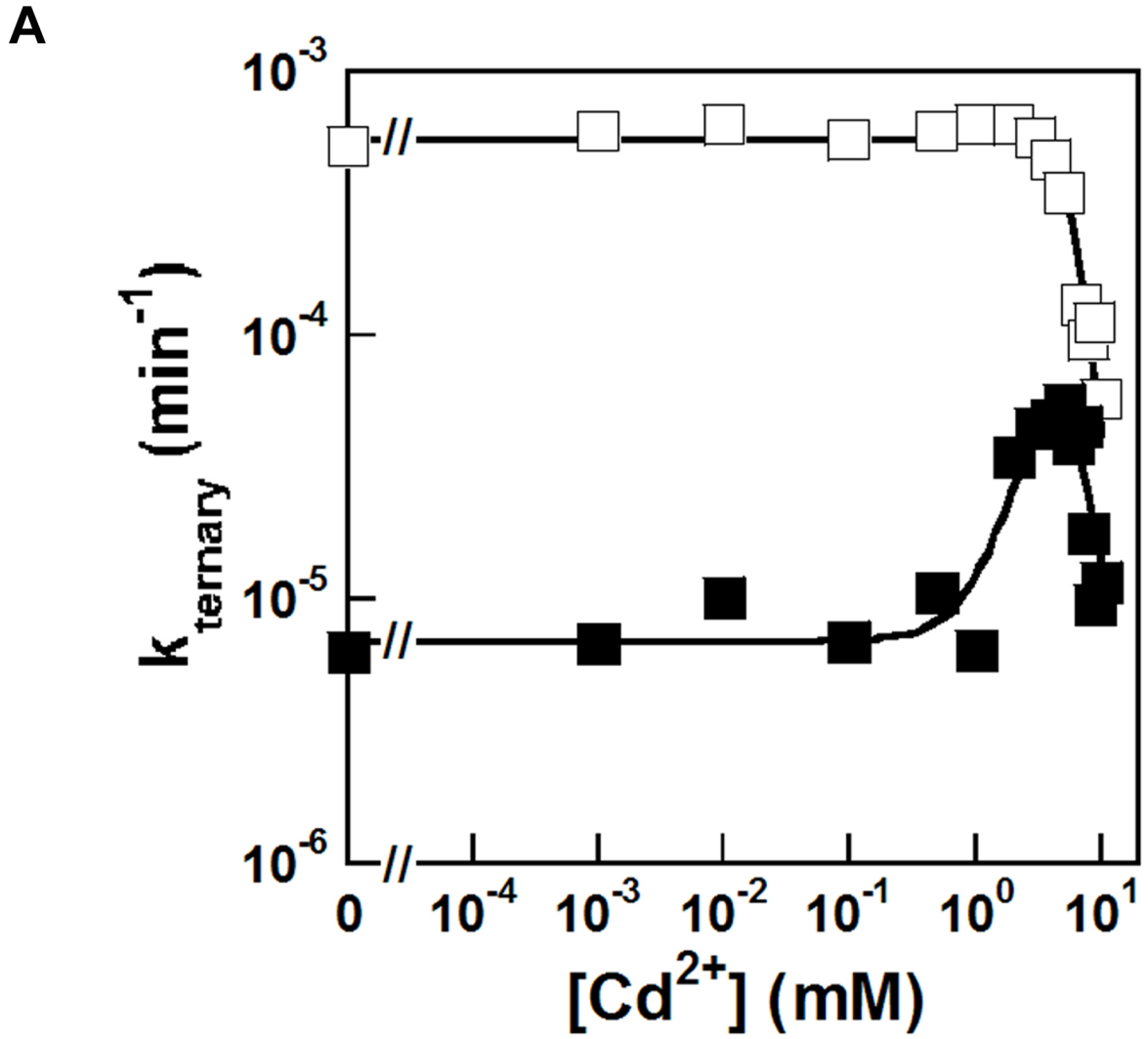

A
10^{-3}
10^{-4}
10^{-5}
10^{-6}
$k_{ternary}$ (min^{-1})
0
10^{-4}
10^{-3}
10^{-2}
10^{-1}
10^{0}
10^{1}
[Cd^{2+}] (mM)

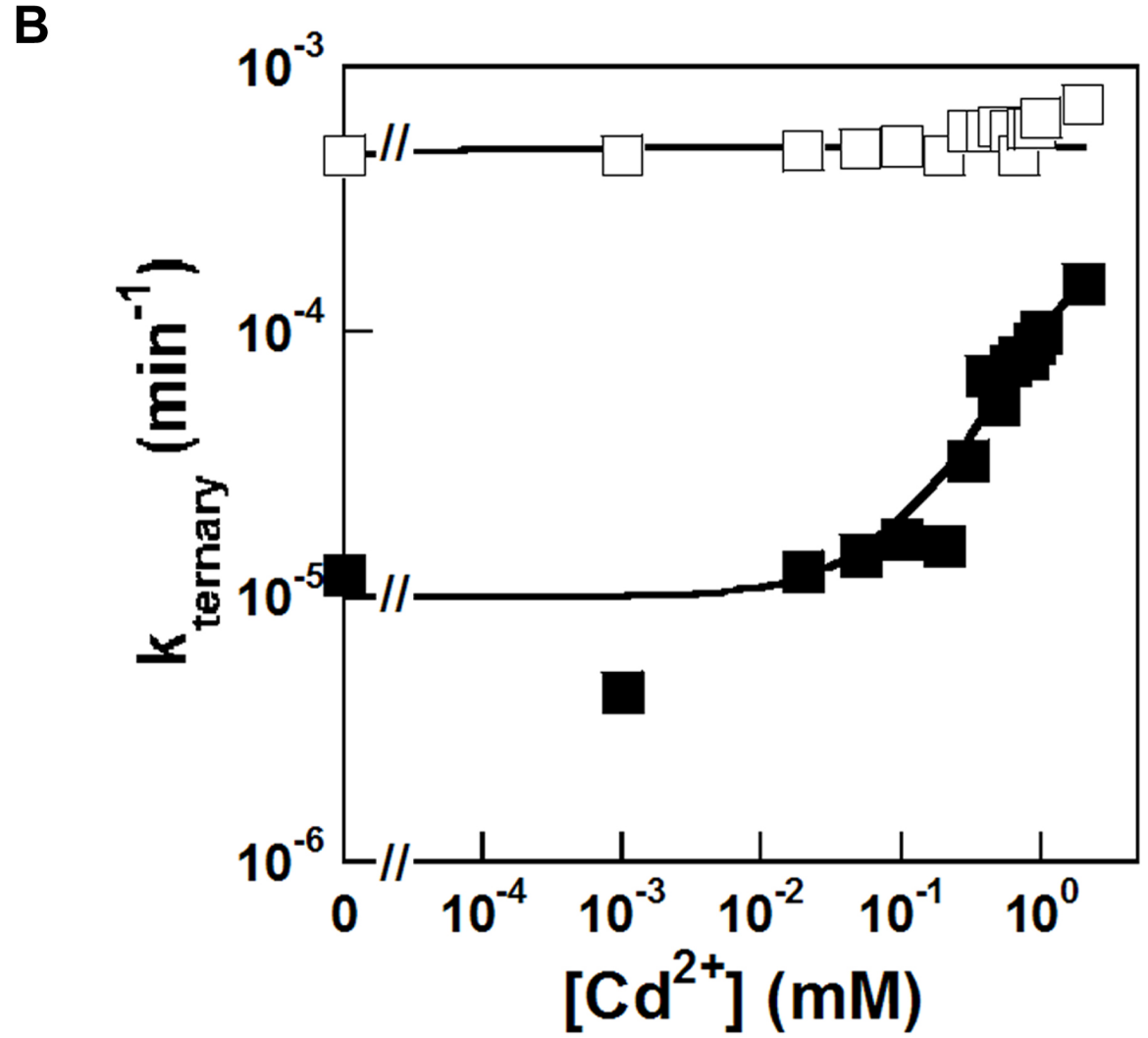

B
10^{-3}
10^{-4}
10^{-5}
10^{-6}
$k_{ternary}$ (min^{-1})
0
10^{-4}
10^{-3}
10^{-2}
10^{-1}
10^{0}
[Cd^{2+}] (mM)
