# Supplementary material for: Functional Identification of Catalytic Metal Ion Binding Sites within RNA
Source: PLoS Biol. 2005 Aug 16;3(9):e277. doi: 10.1371/journal.pbio.0030277 (PMC1184590; doi:10.1371/journal.pbio.0030277)
Supplement: Supplementary file 6 [file pbio.0030277.sd001.doc]

"Functional Identification of Catalytic Metal Ion Binding Sites within RNA"

James L. Hougland, Alexander V. Kravchuk, Daniel Herschlag*, and Joseph A. Piccirilli*

Protocol S1. Analysis of Cd^{2+} rescue profiles

We describe the methods used to fit the Cd^{2+} rescue dependencies for reaction of nitrogen and sulfur modified substrates in the presence of WT and C262-S_P ribozymes. The models and equations presented below follow those from previously reported formulations [1,2].

Cd^{2+} rescue of $S_{m3'S}$ activity

The Cd^{2+} dependence for rescue of $S_{m3'S}$ cleavage relative to -(1-3)d,rSA_5 (Figure 6A) was first fit to a model described by equation s1a, in which the binding of a single Cd^{2+} ion rescues reaction of the 3′-thio substrate. Specific Cd^{2+} rescue of $S_{m3'S}$ cleavage in the presence of both WT and C262-S_P ribozymes was fit to equation s1b, derived from equation s1a, in which k_{obs}^{rel} is the observed relative rate at a given Cd^{2+} concentration, k_{Mg}^{rel} is the relative rate in Mg^{2+} alone, "a" is the observed stimulation of relative cleavage rate upon Cd^{2+} binding at the M_A site (a = k_{Cd}^{rel} / k_{Mg}^{rel}), and $K_{d,app}^{Cd,E•S•G}$ is the apparent affinity for binding of the rescuing Cd^{2+} ion to the E•S•G ternary complex at a given background Mg^{2+} concentration. Rescue of $S_{m3'S}$ observed in the WT ribozyme reaction fit well to this model. The rescue observed in the C262-S_P variant

ribozyme reaction deviated slightly but significantly from this model (data not shown). Cd^{2+} rescue of $S_{m3'S}$ cleavage activity with both WT and C262-S_P was then fit using equation s1c, a modified form of equation s1b that allows for cooperative Cd^{2+} binding, with cooperativity constant n. Rescue with the WT ribozyme fit to this equation with n = 1, consistent with no apparent cooperativity effects on Cd^{2+} binding at metal ion site A. Rescue with the C262-S_P ribozyme fit with n = 1.2, consistent with a small cooperative effect on Cd^{2+} binding or function at metal ion site A for the C262-S_P ribozyme. This small change in cooperativity may result from altered Cd^{2+} binding at the M_C site that slightly perturbs the Cd^{2+} ion at M_A. Nevertheless, the rescue data are still consistent with the rescue of 3′-thio substrate cleavage by a single Cd^{2+} ion binding at metal ion site A. Comparison of Cd^{2+} rescue observed with both WT and C262-S_P ribozymes suggests that the S_P-phosphorothioate substitution at C262 does not significantly alter Cd^{2+} binding and rescue at metal ion site A.

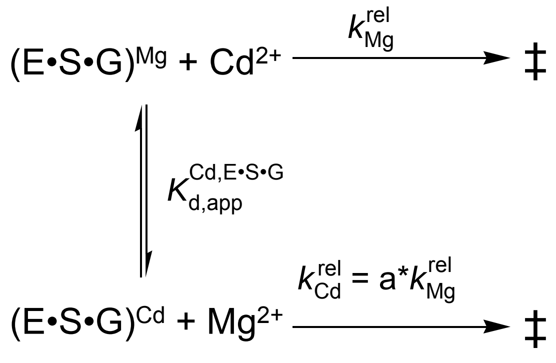

(s1a)

$$k_{obs}^{rel} = k_{Mg}^{rel}\left(\frac{K_{d,app}^{Cd,E\bullet S\bullet G} + a^{*}[Cd^{2+}]}{K_{d,app}^{Cd,E\bullet S\bullet G} + [Cd^{2+}]}\right) \qquad (s1b)$$

$$k_{obs}^{rel} = k_{Mg}^{rel}\left(\frac{(K_{d,app}^{Cd,E\bullet S\bullet G})^n + a^*[Cd^{2+}]^n}{(K_{d,app}^{Cd,E\bullet S\bullet G})^n + [Cd^{2+}]^n}\right) \qquad \text{(s1c)}$$

$CUCG_{3'S}A$ activity rescue by a single Cd^{2+} ion

The Cd^{2+} dependence for reaction of $CUCG_{3'S}A$ relative to CUCGA (Figure 6B) is consistent with the model outlined in equation s2a, with one Cd^{2+} ion binding to the E•P complex and specifically stimulating $CUCG_{3'S}A$ cleavage. The data were fit to equation s2b, derived from equation s2a, in which k_{obs}^{rel} is the observed relative rate at a given Cd^{2+} concentration, k_{Mg}^{rel} is the relative rate in Mg^{2+} alone, "a" is the observed stimulation of relative cleavage rate upon Cd^{2+} binding at the M_B site (a = k_{Cd}^{rel} / k_{Mg}^{rel}), and $K_{d,app}^{Cd,E\bullet P}$ is the apparent Cd^{2+} binding affinity to the E•P complex at the M_B site at a given background Mg^{2+} concentration. Cd^{2+} rescue at site M_B with both WT and C262-S_P ribozymes fits well to this model (Figure 6B) and is unchanged, suggesting that cleavage of $CUCG_{3'S}A$ is specifically rescued by a single Cd^{2+} ion binding at metal ion site B in both ribozymes.

$$\begin{array}{lcl} (E\bullet P)^{Mg} + CUCG_{3'X}A + Cd^{2+} & \xrightarrow{k_{Mg}^{rel}} & \ddagger \\ \quad \Big\updownarrow K_{d,app}^{Cd,E\bullet P} & & \\ (E\bullet P)^{Cd} + CUCG_{3'X}A + Mg^{2+} & \xrightarrow{k_{Cd}^{rel} = a^*k_{Mg}^{rel}} & \ddagger \end{array} \qquad \text{(s2a)}$$

$$k_{obs}^{rel} = k_{Mg}^{rel}\left(\frac{K_{d,app}^{Cd,E•P}}{K_{d,app}^{Cd,E•P} + [Cd^{2+}]}\right) + a*k_{Mg}^{rel}\left(\frac{[Cd^{2+}]}{K_{d,app}^{Cd,E•P} + [Cd^{2+}]}\right) \quad (s2b)$$

Rescue of G_N activity by a single Cd^{2+} ion under $(k_c/K_m)^{G\ (or\ G_N)}$ conditions

The Cd^{2+} dependence for oligonucleotide substrate (S) cleavage by G_N relative to cleavage by G, measured under subsaturating G or G_N conditions [$(k_c/K_m)^{G\ (or\ G_N)}$ conditions] (Figure 6C), is consistent with the model outlined in equation s3a, in which the binding of one Cd^{2+} ion to the E•S complex specifically stimulates cleavage by G_N. The data were fit to equation s3b, derived from equation s3a, in which k_{obs}^{rel} is the observed relative rate at a given Cd^{2+} concentration, k_{Mg}^{rel} is the relative rate in Mg^{2+} alone, “a” is the observed stimulation of relative cleavage rate upon Cd^{2+} binding at the M_C site ($a = k_{Cd}^{rel} / k_{Mg}^{rel}$), and $K_{d,app}^{Cd,E•S}$ is the apparent Cd^{2+} binding affinity to the E•S complex at the M_C site at a given background Mg^{2+} concentration. The Cd^{2+} dependence of k_{obs}^{rel} fit well to this model for reactions catalyzed by both the WT and C262-S_P ribozymes, consistent with specific stimulation of S cleavage by G_N from a single Cd^{2+} ion binding at metal ion site C in both ribozymes (Figure 6C).

$$(E\bullet S)^{Mg} + G\ (\text{or}\ G_N) + Cd^{2+} \xrightarrow{k^{rel}_{Mg}} \ddagger$$

$$\Big\Updownarrow\ K^{Cd,\ E\bullet S}_{d,app} \qquad \text{(s3a)}$$

$$(E\bullet S)^{Cd} + G\ (\text{or}\ G_N) + Mg^{2+} \xrightarrow{k^{rel}_{Cd} = a^{*}k^{rel}_{Mg}} \ddagger$$

$$k^{rel}_{obs} = k^{rel}_{Mg}\left(\frac{K^{Cd,\ E\bullet S}_{d,app}}{K^{Cd,\ E\bullet S}_{d,app} + [Cd^{2+}]}\right) + a^{*}k^{rel}_{Mg}\left(\frac{[Cd^{2+}]}{K^{Cd,\ E\bullet S}_{d,app} + [Cd^{2+}]}\right) \qquad \text{(s3b)}$$

Rescue of G_N activity by a single Cd^{2+} ion under $(E\bullet S\bullet G/G_N)_O \rightarrow$ products ($k_{ternary}$) conditions

The Cd^{2+} dependence for S cleavage by G_N relative to cleavage by G under $(E\bullet S\bullet G/G_N)_O \rightarrow$ products ($k_{ternary}$) conditions (Figure 6D) is consistent with the model outlined in equation s4a, in which the binding of one Cd^{2+} ion to the $E\bullet S\bullet G/G_N$ ternary complex specifically stimulates cleavage by G_N. The data were fit to equation s4b, derived from equation s4a, in which k^{rel}_{obs} is the observed relative rate at a given Cd^{2+} concentration, k^{rel}_{Mg} is the relative rate in Mg^{2+} alone, "a" is the observed stimulation of relative cleavage rate upon Cd^{2+} binding at the M_C site ($a = k^{rel}_{Cd} / k^{rel}_{Mg}$), and $K^{Cd, E\bullet S\bullet G/G_N}_{d,app}$ is the apparent Cd^{2+} binding affinity to the $E\bullet S\bullet G/G_N$ ternary complex at the M_C site at a given background Mg^{2+}

concentration. The Cd^{2+} dependence of k_{obs}^{rel} fit well to this model for reactions catalyzed by both WT and C262-S_P ribozymes, consistent with specific stimulation of G_N cleavage of S from a single Cd^{2+} ion binding at the M_C site in both ribozymes.

$$\begin{array}{ccc} (E\bullet S\bullet G/G_N)_O^{Mg} + Cd^{2+} & \xrightarrow{k_{Mg}^{rel}} & \ddagger \\ \Big\Updownarrow K_{d,app}^{Cd,\,E\bullet S\bullet G/G_N} & & \\ (E\bullet S\bullet G/G_N)_O^{Cd} + Mg^{2+} & \xrightarrow{k_{Cd}^{rel} = a^*k_{Mg}^{rel}} & \ddagger \end{array} \qquad \text{(s4a)}$$

$$k_{obs}^{rel} = k_{Mg}^{rel}\left(\frac{K_{d,app}^{Cd,\,E\bullet S\bullet G/G_N}}{K_{d,app}^{Cd,\,E\bullet S\bullet G/G_N} + [Cd^{2+}]}\right) + a^*k_{Mg}^{rel}\left(\frac{[Cd^{2+}]}{K_{d,app}^{Cd,\,E\bullet S\bullet G/G_N} + [Cd^{2+}]}\right) \qquad \text{(s4b)}$$

Rescue of S_{Sp} substrate activity by two Cd^{2+} ions under $(E\bullet S\bullet G/G_N)_O \rightarrow$ products ($k_{ternary}$) conditions

We determined the Cd^{2+} dependence for C262-S_P ribozyme catalyzed cleavage of the S_{Sp} substrate with both G and G_N relative to the reaction of the -(1-3)d,rSA_5 substrate with G (Figures 7B and 7D, respectively). Reactions were monitored under $(E\bullet S\bullet G/G_N)_O \rightarrow$ products ($k_{ternary}$) conditions. The data are consistent with the model depicted in equation s5a, in which two Cd^{2+} ions bind to the ribozyme at the metal ion sites A and C to specifically stimulate S_{Sp} substrate cleavage. In

this model, $(E{\bullet}S{\bullet}G/G_N)^{Mg_A}_{Mg_C}$ is ribozyme with Mg^{2+} bound at both metal ion sites A and C, $(E{\bullet}S{\bullet}G/G_N)^{Cd_A}_{Mg_C}$ is ribozyme with Cd^{2+} bound at metal ion site A and Mg^{2+} bound at metal ion site C, $(E{\bullet}S{\bullet}G/G_N)^{Mg_A}_{Cd_C}$ is ribozyme with Mg^{2+} bound at metal site A and Cd^{2+} bound at metal ion site C, and $(E{\bullet}S{\bullet}G/G_N)^{Cd_A}_{Cd_C}$ is ribozyme with Cd^{2+} bound at both metal ion sites. This model assumes that no stimulation of S_{Sp} cleavage activity occurs unless Cd^{2+} ions occupy both the metal ion binding sites, and that Cd^{2+} affinities at these two sites are independent of one another. More complex models are possible as described by Wang et al., but equation s5a provides the simplest model that accounts quantitatively for the data [3]. The data were fit to equation s5b, derived from equation s5a, in which k^{rel}_{obs} is the observed relative rate at a given Cd^{2+} concentration, k^{rel}_{Mg} is the relative rate in Mg^{2+} alone, a is the observed stimulation of relative cleavage rate upon Cd^{2+} binding at both metal ion binding sites, and $K^{Cd,M_A}_{d,app}$ and $K^{Cd,M_C}_{d,app}$ are the apparent Cd^{2+} binding affinities to the $E{\bullet}S{\bullet}G/G_N$ complex at metal ion sites A and C, respectively, in a given Mg^{2+} background concentration.

For cleavage of S_{Sp} in the presence of saturating G_N, $K^{Cd,M_C}_{d,app}$ was fixed at 0.2 mM, the apparent Cd^{2+} affinity observed at metal ion site C during Cd^{2+} rescue of substrate cleavage by G_N under $k_{ternary}$ conditions (Figure 6D). The Cd^{2+} rescue profile for cleavage of S_{Sp} by the C262-Sp ribozyme in the presence of bound G_N partially supports the assumption that no stimulation of S_{Sp} cleavage occurs unless Cd^{2+} occupies both the M_A and M_C binding sites. In the presence of bound G_N, the S_{Sp} reaction exhibits significant stimulation only at Cd^{2+}

concentrations near $K_{d,app}^{Cd,M_C}$ = 0.2 mM (cf. Figure 6D), suggesting that the $(E{\bullet}S{\bullet}G/G_N)^{Mg_A}_{Cd_C}$ complex reacts no faster than the $(E{\bullet}S{\bullet}G/G_N)^{Mg_A}_{Mg_C}$ complex. As discussed in the following section, alternative models for stimulation of the S_{Sp} reaction by a single Cd^{2+} ion also appear unlikely.

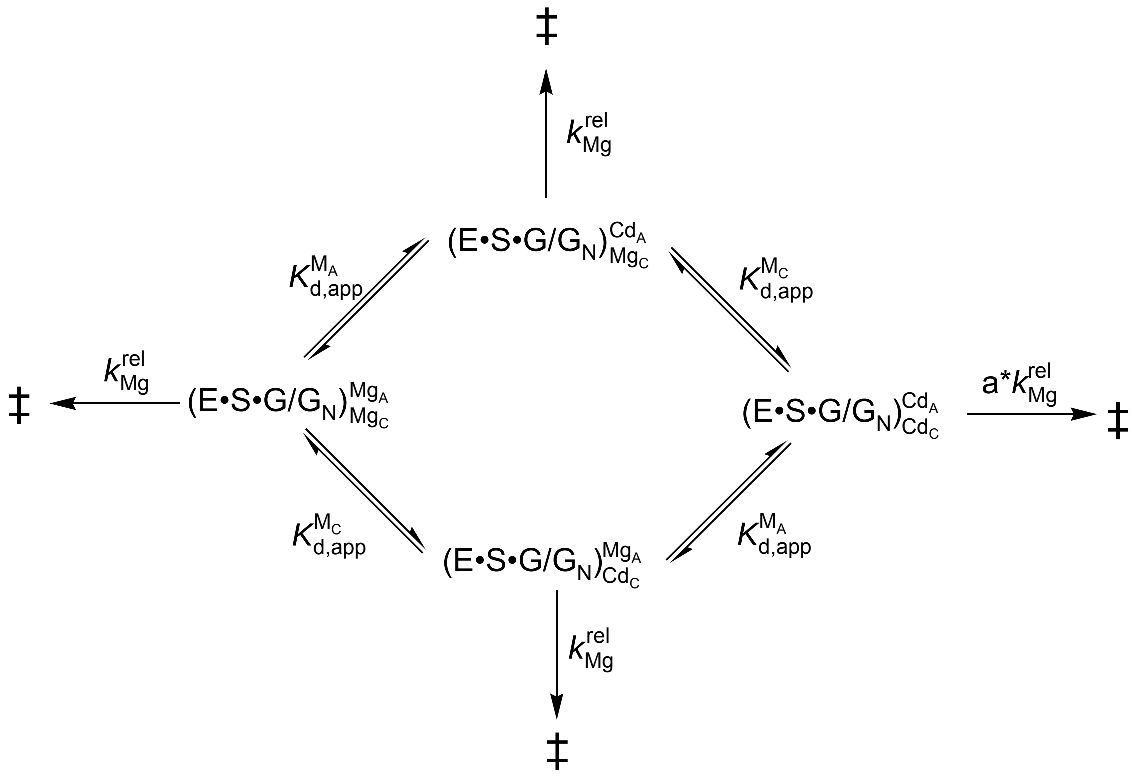

(s5a)

$$k_{obs}^{rel} = k_{Mg}^{rel} \left(\frac{1 + \frac{[Cd^{2+}]}{K_{d,app}^{M_A}} + \frac{[Cd^{2+}]}{K_{d,app}^{M_C}} + a^* \frac{[Cd^{2+}]^2}{K_{d,app}^{M_A} K_{d,app}^{M_C}}}{1 + \frac{[Cd^{2+}]}{K_{d,app}^{M_A}} + \frac{[Cd^{2+}]}{K_{d,app}^{M_C}} + \frac{[Cd^{2+}]^2}{K_{d,app}^{M_A} K_{d,app}^{M_C}}} \right) \quad \text{(s5b)}$$

Alternative models for the change in Cd^{2+} dependence of S_{Sp} substrate rescue in the presence of saturating G_N and the C262-S_P ribozyme

Two other models to explain the change in Cd^{2+} dependence of S_{Sp} cleavage upon C262-S_P mutation require consideration. First, the C262-S_P phosphorothioate could perturb the ribozyme active site sufficiently as to allow only one metal ion, M_A or M_C, to coordinate to the substrate S_P-phosphorothioate mutation in the transition state, resulting in a Cd^{2+} rescue profile that reflects only one rescuing metal ion. However, this model is inconsistent with the rescue by two Cd^{2+} ions exhibited by the S_{Sp} reaction in the presence of saturating guanosine, assuming the G and G_N-bound ribozyme active sites are identical except for the 2′-OH → 2′-NH_2 modification (a change with minimal steric consequences and minimal functional effects in the WT reaction [4,5]).

Second, the Cd^{2+} rescue observed for S_{Sp} cleavage in the presence of saturating G_N could reflect only M_C rescue of the G_N-induced reactivity defect, without any rescue of the S_P phosphorothioate mutation itself. If this model were valid, the Cd^{2+} rescue profile observed for S_{Sp} cleavage by saturating G_N should be identical to the profile obtained during Cd^{2+} rescue of G_N cleavage of the -1r,dSA$_5$ substrate under $k_{ternary}$ conditions, i.e. sat′urating G_N (Figure 6D), with

rescue reaching a plateau above 1 mM Cd^{2+} as metal ion site C saturates with an apparent Cd^{2+} binding affinity of ~0.2 mM. However, Cd^{2+} rescue of S_{Sp} cleavage activity in the presence of saturating G_N increases linearly up to 1 mM Cd^{2+}, inconsistent with this stimulation reflecting Cd^{2+} binding at only metal ion site C. This continued increase in S_{Sp} activity is consistent with rescue requiring Cd^{2+} ion binding at both a high affinity site that saturates at low Cd^{2+} concentration (metal ion site C) and a low-affinity Cd^{2+} ion binding site (metal ion site A) that does not saturate within the experimentally accessible Cd^{2+} concentration range.

1 Shan S, Yoshida A, Sun SG, Piccirilli JA, Herschlag D (1999) Three metal ions at the active site of the *Tetrahymena* group I ribozyme. Proc Natl Acad Sci USA 96: 12299-12304.

2 Shan S, Kravchuk AV, Piccirilli JA, Herschlag D (2001) Defining the catalytic metal ion interactions in the *Tetrahymena* ribozyme reaction. Biochemistry 40: 5161-5171.

3 Wang SL, Karbstein K, Peracchi A, Beigelman L, Herschlag D (1999) Identification of the hammerhead ribozyme metal ion binding site responsible for rescue of the deleterious effect of a cleavage site phosphorothioate. Biochemistry 38: 14363-14378.

4 Shan SO, Herschlag D (1999) Probing the role of metal ions in RNA catalysis: Kinetic and thermodynamic characterization of a metal ion interaction with the 2'-moiety of the guanosine nucleophile in the *Tetrahymena* group I ribozyme. Biochemistry 38: 10958-10975.

5 Sjogren AS, Pettersson E, Sjoberg BM, Stromberg R (1997) Metal ion interaction with cosubstrate in self-splicing of group I introns. Nucleic Acids Res 25: 648-653.
