# Supplementary material for: Functional Identification of Catalytic Metal Ion Binding Sites within RNA
Source: PLoS Biol. 2005 Aug 16;3(9):e277. doi: 10.1371/journal.pbio.0030277 (PMC1184590; doi:10.1371/journal.pbio.0030277)
Supplement: Supplementary file 7 [file pbio.0030277.sd002.doc]

**“Functional Identification of Catalytic Metal Ion Binding Sites within RNA”**

James L. Hougland, Alexander V. Kravchuk, Daniel Herschlag*, and Joseph A. Piccirilli*

**Protocol S2. Measuring oligonucleotide substrate association and dissociation rate constants**

Oligonucleotide substrate (S) association was measured at several ribozyme concentrations (1-10 nM). Radiolabeled S (*S, -1d,rSA_5_, <1 nM) was added to ribozyme; aliquots were withdrawn at six time points and carefully loaded onto a running 8% native THEM polyacrylamide gel (33 mM Tris, 67 mM HEPES, 1 mM EDTA, 10 mM MgCl_2_) [1]. E•S complex migrated as a high molecular weight band and free S migrated as a lower molecular weight band. Observed association rate constants were measured by quantitating the partial population of bound versus unbound substrate as a function of time. In control reactions, addition of excess unlabeled S prior to addition of *S blocked binding of *S. The second order association rate constant, *k*_on_, was measured as the slope of observed association rate versus ribozyme concentration.

Dissociation rate constants were measured using the same method as reported previously [2,3]. Radiolabeled substrate (*S, <1 nM) was incubated with saturating ribozyme [100 nM] for 5 minutes, and then a large excess of unlabeled S (1-2 μM) was added. Aliquots were withdrawn at six time points and carefully loaded onto a running 8% native THEM polyacrylamide gel (33 mM Tris, 67 mM HEPES, 1 mM EDTA, 10 mM MgCl_2_) [1]. Dissociation rate constants were measured by quantitating the partial population of unbound versus bound substrate as a function of time. In control reactions, addition of excess unlabeled S prior to addition of *S blocked binding of *S.

1	Mei R, Herschlag D (1996) Mechanistic investigations of a ribozyme derived from the *Tetrahymena* group I intron: Insights into catalysis and the second step of self-splicing. Biochemistry 35: 5796-5809.

2	Karbstein K, Carroll KS, Herschlag D (2002) Probing the *Tetrahymena* group I ribozyme reaction in both directions. Biochemistry 41: 11171-11183.

3	Herschlag D, Cech TR (1990) Catalysis of RNA cleavage by the *Tetrahymena* *thermophila* ribozyme. 1. Kinetic description of the reaction of an RNA substrate complementary to the active site. Biochemistry 29: 10159-10171.
