# Supplementary material for: Functional Identification of Catalytic Metal Ion Binding Sites within RNA
Source: PLoS Biol. 2005 Aug 16;3(9):e277. doi: 10.1371/journal.pbio.0030277 (PMC1184590; doi:10.1371/journal.pbio.0030277)
Supplement: Supplementary file 8 [file pbio.0030277.st001.doc]

**“Functional Identification of Catalytic Metal Ion Binding Sites within RNA”**

James L. Hougland, Alexander V. Kravchuk, Daniel Herschlag*, and Joseph A. Piccirilli*

**Table S1.** Oligonucleotide substrate binding to WT and C262-*S*_P_ ribozymes^a^

| Ribozyme | *k*_on_ (10^8^ M^-1^min^-1^)^b^ | *k*_off_ (min^-1^)^b^ | | *K*${}_{d(E)}^{S}$ (nM)^d^ | |
| --- | --- | --- | --- | --- | --- |
|  |  | -1d,rSA_5_ | -(1-3)d,rSA_5_^c^ | -1d,rSA_5_ | -(1-3)d,rSA_5_^c^ |
| WT | 1.1 ± 0.09 | 0.021 ± 0.006 | 0.59 ± 0.06 | 0.2 | 5 |
| C262-*S*_P_ | 1.0 ± 0.1 | 0.042 ± 0.01 | 0.58 ± 0.08 | 0.4 | 6 |

^a^ Binding was measured in the presence of 10 mM MgCl^­^_2_ without added Cd^2+^. Addition of 0.1 mM CdCl_2_ did not appreciably affect measured association or dissociation rate constants (data not shown).

^b^ *k*_on_ and *k*_off_ were measured as described in *Materials and Methods*.

^c^ Reactions with -(1-3)d,rSA_5_ probe effects on substrate binding to the open complex,(E•S)_O_ (Figure 3).

^d^ *K*${}_{d(E)}^{S}$ was calculated by *K*${}_{d(E)}^{S}$= $\frac{k_{\text{off}}}{k_{\text{on}}}$ . *K*${}_{d(E)}^{S}$for -1d,rSA_5_ reflects S binding affinity in the closed complex (Figure 3). *K*${}_{d(E)}^{S}$for -(1-3)d,rSA_5_ reflects S binding affinity in the open complex (Figure 3).
