# Supplementary material for: Functional Identification of Catalytic Metal Ion Binding Sites within RNA
Source: PLoS Biol. 2005 Aug 16;3(9):e277. doi: 10.1371/journal.pbio.0030277 (PMC1184590; doi:10.1371/journal.pbio.0030277)
Supplement: Supplementary file 9 [file pbio.0030277.st002.doc]

**“Functional Identification of Catalytic Metal Ion Binding Sites within RNA”**

James L. Hougland, Alexander V. Kravchuk, Daniel Herschlag*, and Joseph A. Piccirilli*

**Table S2.** G binding and reactivity of WT ribozymes constructed by transcription and ligation^a^

| Ribozyme | *K*${}_{d(E\cdot S)_{C}}^{G}$ (μM) | k_c_ (min^-1^) |
| --- | --- | --- |
| Transcribed | 96 ± 16 | 0.038 ± 0.002 |
| Ligated | 82 ± 18 | 0.025 ± 0.003 |

^a^ Cleavage of -1d,rSA_5_ (Table 1) was followed as a function of guanosine concentration. Reactions were performed with saturating ribozyme (100 nM), 10 mM MgCl_2_, 50 mM NaMOPS, pH 7.0, and 30 ^o^C.
